# The Association with Subclinical Thyroid Dysfunction and Uric Acid

**DOI:** 10.1155/2021/9720618

**Published:** 2021-12-13

**Authors:** Yuling Xing, Linlin Yang, Jing Liu, Huijuan Ma

**Affiliations:** ^1^Department of Endocrinology, Hebei General Hospital, Shijiazhuang 050017, China; ^2^Graduate School of Hebei Medical University, Shijiazhuang 050017, China; ^3^Hebei Key Laboratory of Metabolic Diseases, Hebei General Hospital, Shijiazhuang, Hebei 050051, China; ^4^Department of Internal Medicine, Hebei Medical University, Shijiazhuang, Hebei 050017, China

## Abstract

The relationship between subclinical thyroid dysfunction and uric acid was not well established. This study aimed to determine if subclinical thyroid dysfunction is associated with hyperuricemia risk and to evaluate the levels of uric acid in patients with different forms of subclinical thyroid dysfunction. A systematic search was conducted in 4 databases to obtain relevant studies on subclinical thyroid dysfunction (subclinical hyperthyroidism and subclinical hypothyroidism) and uric acid. The standardized mean difference (SMD) or odds ratio (OR) and 95% confidence interval (95% CI) were used for evaluation, and the sensitivity analysis was conducted. Publication bias was estimated by funnel plot, Egger's test, and Begg's test. A total of 73 studies were included in this meta-analysis. The results demonstrated that serum levels of uric acid in patients with subclinical hypothyroidism were significantly higher than those of controls and patients with subclinical hyperthyroidism. Patients with subclinical thyroid dysfunction had a higher prevalence of hyperuricemia compared with normal clinical thyroid function. Subclinical thyroid dysfunction was associated with the prevalence of hyperuricemia. Different types of subclinical thyroid dysfunction had varied effects on serum levels of uric acid.

## 1. Introduction

Thyroid hormones elicited significant effects on numerous physiological processes, such as growth, development, and metabolism. Thyroid dysfunction is a common endocrine disease and consists of overt hypothyroidism (OH), subclinical hypothyroidism (SCH), overt hyperthyroidism (OHyper), and subclinical hyperthyroidism (SCHyper). Subclinical thyroid dysfunction was characterized by high (SCH)/low (SCHyper) TSH concentrations and normal serum thyroid hormones or serum-free thyroid hormones [1, 2]. The prevalence of SCH was approximately 4–10% [3, 4], and it can be as high as 20% in people over 60 years old [5]. SCHyper was also a common thyroid disorder with a prevalence of up to 10% [6–8]. Subclinical thyroid dysfunction, which can be diagnosed by thyroid function tests before symptoms and complications occur, is viewed as a risk factor for developing hyperthyroidism and hypothyroidism complications [9]. Moreover, a growing body of observational data suggests that cardiovascular risk may also be increased in subgroups of patients with SCH or SCHyper [10]. Uric acid (UA) is the end product of the purine metabolism in the human body. Serum UA levels reflected a balance between the metabolic breakdown of purine nucleotides and UA excretion [11]. Serum UA levels have been considered as an independent predictive factor for metabolic syndrome [12, 13]. Hyperuricemia is the best-known risk factor for gout, but it is also a risk factor for hypertension, diabetes, and chronic kidney disease (CKD) [14–16]. Across the globe, hyperuricemia was becoming a critical medical problem, and its prevalence has dramatically increased in past decades [17, 18]. Many epidemiologic studies have suggested that hyperuricemia is associated with hypertension, cardiovascular diseases, diabetes mellitus, and dyslipidemia [19–23]. Uric acid as the end product of the purine metabolism can be affected by thyroid hormones. Therefore, we hypothesized that a link between UA and thyroid function may exist. Although previous studies have investigated the association between overt thyroid dysfunction and UA [24–27], the results are quite inconsistent between subclinical thyroid dysfunction and UA. A recent study by YE Y et al. showed that subclinical thyroid dysfunction was not significantly associated with serum UA levels, either SCHyper or SCH [28]. Zhang et al. found the marked elevated risk of hyperuricemia observed among the subjects with SCH [29]. This study, therefore, aimed to evaluate the association between subclinical thyroid dysfunction and hyperuricemia and focus on variation in subclinical thyroid dysfunction styles and serum UA levels.

## 2. Materials and Methods

### 2.1. Literature Search

We adopted PubMed, Embase, Cochrane library, and China Academic Journal Full-text Database (CNKI) to search relevant literature before March 2021. A systematic search for “subclinical hyperthyroidism/hypothyroidism” and “hyperuricemia/uric acid” was carried out. Key-terms were grouped and searched within the article title, abstract, and keywords using the conjunctions “OR” and “AND.” Selection of studies: after initial screening of titles and abstracts retrieved by the search, the full text of all potentially eligible studies was retrieved.

### 2.2. Inclusion Criteria

The inclusion criteria were as follows: the study was an observational or prospective study; data provided within the study met the needs to confirm the relationship between subclinical thyroid dysfunction and uric acid; the control group was included in the study or data for before and after therapy of subclinical thyroid dysfunction; there was no direct associations among studies; and the patient was diagnosed as subclinical hyperthyroidism, subclinical hypothyroidism, and hyperuricemia by a clear diagnosis.

### 2.3. Exclusion Criteria

The exclusion criteria were as follows: Animal studies, reviews, and case reports; studies that used data from a previously published study; and the data within the study which were not complete enough to meet the requirements of meta-analysis.

### 2.4. Literature Screening and Data Extraction

The first stage involved screening titles and abstracts to identify and exclude irrelevant articles. All full-text versions of studies that were potentially relevant were then screened in relation to the inclusion criteria. Two researchers independently searched and screened the literature and collected and cross-checked the relevant data. If the results were inconsistent, those would be discussed together or judged by a third senior researcher. Data from included studies were extracted and summarized independently using a prestandardized data extraction form. The excerpts included basic characteristics (year of publication, study area, number of participants, diagnostic criteria, the determination method of UA and thyroid hormones, and inclusion and exclusion criteria). Mean ± SD was extracted when the level of UA was used as a continuous variable, and the corresponding proportion was extracted when the level of UA was used as a binary variable. The cutoff value for the diagnosis of hyperuricemia and subclinical thyroid dysfunction was extracted.

### 2.5. Statistical Analysis

The data and the database were organized and checked carefully according to the requirements of the meta-analysis. The RevMan 5.3 analysis software was used for statistical analysis. Standardized mean difference (SMD) for continuous variables, with 95% confidence interval (CI), was calculated for each study. For analyses of dichotomous variables, we used risk ratios (OR) and 95% confidence intervals (95% CI). The *Z*-test was assessed to evaluate the significance of the pooled effect size. If *I*^2^ ≤ 50% or *P* ≥ 0.05, fixed effect model analysis was used; if *I*^2^ > 50% or *P* < 0.05, random effect model analysis was used. The sensitivity analysis was tested to determine the stability and reliability of the results in this meta‐analysis. In addition, we will run subgroup analysis to explore possible sources of obvious heterogeneity. Funnel plot, Egger's test, and Begg's test were used to evaluate publication bias. *P* < 0.05 was considered statistically significant, suggesting that publication bias is not excluded. The stability of the conclusions was further evaluated after eliminating publication bias by the trim-and-fill method. Meta-regression and subgroup analysis were performed to explore the source of heterogeneity.

## 3. Result

### 3.1. Literature Search Results

**Figure 1:**
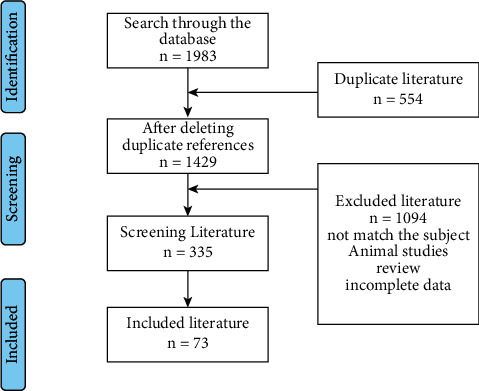
Literature screening process and results.

The systematic literature search retrieved 1983 publications; after exclusion of duplicates and screening for relevance in title and abstract, 1429 publications were further appraised in full text. In the second step, full texts were reviewed for eligibility and relevance of their findings, and 1094 articles were excluded due to duplicate data, review articles, and insufficient relevance. Finally, a total of 73 articles were included in the meta-analysis (Figure 1). We did not exclude any studies in the review based on the comorbidities of the study participants, but we kept into account this aspect when summarizing the results. Supplementary Materials (Table S1) provide the basic characteristics of included studies.

### 3.2. Meta-Analysis Results

#### 3.2.1. Relationship between SCH/SCHyper and the Prevalence of Hyperuricemia

**Figure 2:**
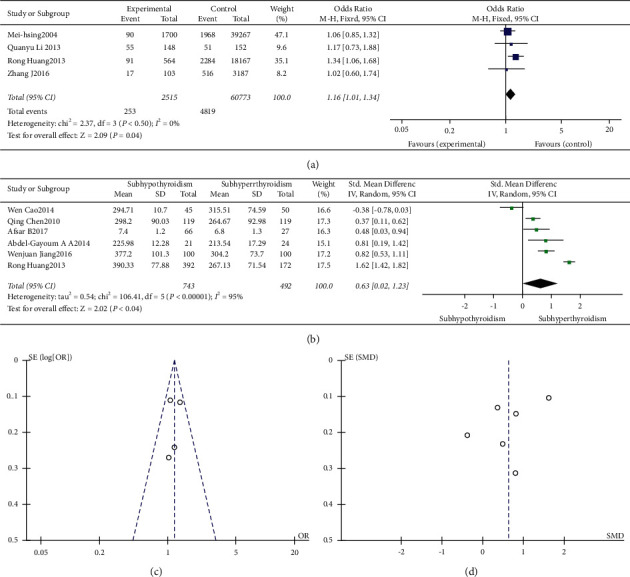
(a) Forest plot of subclinical thyroid dysfunction and hyperuricemia. (b) Forest plot of the comparison of subclinical hypothyroidism and subclinical hyperthyroidism. (c) Funnel plot of subclinical thyroid dysfunction and hyperuricemia. (d) Funnel plot of the comparison of subclinical hypothyroidism and subclinical hyperthyroidism.

A total of 4 studies provided a comparison of the prevalence of hyperuricemia. Among them, 2 studies were related to the comparison of the prevalence of hyperuricemia in SCHyper patients and normal thyroid function individuals. 3 studies involved the comparison of the prevalence of hyperuricemia between SCH patients and normal thyroid function people. It was shown that the prevalence of hyperuricemia of patients with subclinical thyroid dysfunction was higher than that of subjects with normal thyroid function, and the difference was statistically significant (*I*^2^ = 0%, *P* = 0.50, *Z* = 2.09, *P* = 0.04, OR = 1.16, 95% CI: 1.01–1.34, Figure 2(a)).

#### 3.2.2. Relationship between SCH/SCHyper and Serum UA Levels

**Figure 3:**
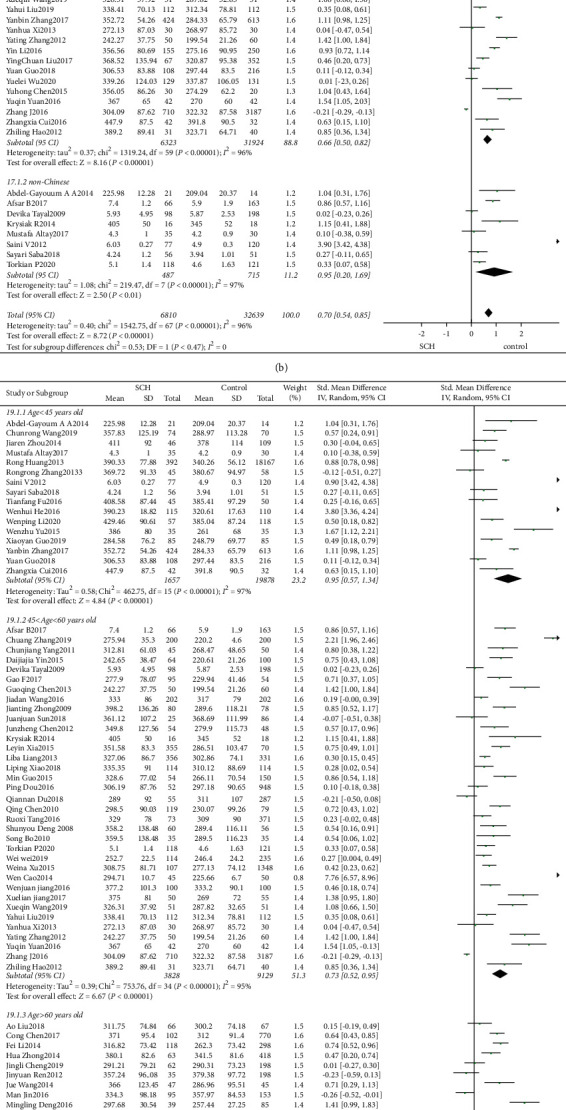
(a) Forest plot of subclinical hypothyroidism and uric acid. (b) Subgroup analysis of subclinical hypothyroidism, grouped by area. (c) Subgroup analysis of subclinical hypothyroidism, grouped by age. (d) Subgroup analysis of subclinical hypothyroidism, grouped by basic diseases. (e) Funnel plot of subclinical hypothyroidism.

**Figure 4:**
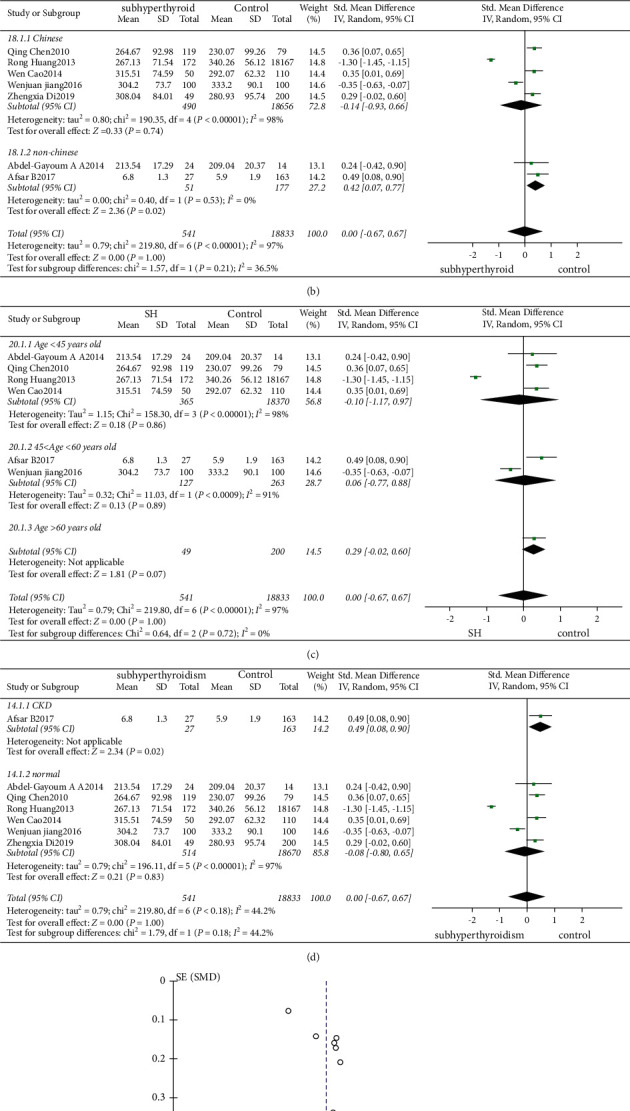
(a) Forest plot of subclinical hyperthyroidism and uric acid. (b) Subgroup analysis of subclinical hyperthyroidism, grouped by area. (c) Subgroup analysis of subclinical hyperthyroidism, grouped by age. (d) Subgroup analysis of subclinical hyperthyroidism, grouped by basic diseases. (e) Funnel plot of subclinical hyperthyroidism.

68 studies involved the comparison of serum UA levels between patients with SCH and subjects with normal thyroid function. 7 studies involved the comparison of serum UA levels between patients with SCHyper and subjects with normal thyroid function. 6 studies involved the comparison of serum UA levels between patients with SCH and SCHyper. The results showed that serum UA levels were significantly higher in patients with SCH than in those of normal controls (*I*^2^ = 96%, *P* < 0.01, *Z* = 9.04, *P* < 0.01, SMD = 0.78, 95% CI: 0.61–0.95, Figure 3(a)). There was no statistical difference in the levels of UA between patients with SCHyper and normal controls (*I*^2^ = 97%, *P* < 0.01, *Z* = 0.00, *P* = 1.00, SMD = 0.00, 95% CI: −0.67–0.67, Figure 4(a)). In addition, levels of UA in patients with SCH were significantly higher than those with SCHyper (*I*^2^ = 95%, *P* < 0.01, *Z* = 2.02, *P* = 0.04, SMD = 0.63, 95% CI: 0.02–1.23, Figure 2(b)).

#### 3.2.3. Meta-Regression

Meta-regression analysis showed that patient age (*P* = 0.076) and TSH level (*P* = 0.608) did not significantly impact the UA level in patients with SCH compared with those with normal thyroid function. However, area (*P* = 0.004) affected the pooled effect size.

#### 3.2.4. Subgroup Analysis

Due to the heterogeneity of the studies included, in order to further increase the reliability of the study, a subgroup analysis of age, area, and comorbidities in patients was performed.

1. Area: (a) SCH: according to the area, patients were divided into two subgroups: Chinese and non-Chinese. There were 60 studies involving 38247 subjects in Chinese (*I*^2^ = 96%, *P* < 0.01, *Z* = 8.16, *P* < 0.01, SMD = 0.66, 95% CI: 0.50–0.82), and 8 studies involving 1202 subjects elsewhere (*I*^2^ = 97%, *P* < 0.01, *Z* = 2.50, *P* < 0.01, SMD = 0.95, 95% CI: 0.20–1.69). These outcomes suggested the UA levels were higher in SCH patients, regardless of whether the patients were Chinese, and the difference was statistically significant (Figure 3(b)). (b) SCHyper: there were 5 studies involving 19146 subjects in Chinese (*I*^2^ = 98%, *P* < 0.01, *Z* = 0.33, *P* = 0.74, SMD = −0.14, 95% CI: −0.93–0.66), and 2 studies involving 228 subjects elsewhere (*I*^2^ = 0%, *P* = 0.53, *Z* = 2.360, *P* = 0.02, SMD = 0.42, 95% CI: 0.07–0.77). These outcomes suggested UA levels were higher in SCHyper patients only in those who were non-Chinese (Figure 4(b)).
2. Age: according to the average age of patients with subclinical thyroid dysfunction, the subjects were divided into three subgroups: age < 45 years old, 45 ≤ age < 60 years old, and age ≥ 60 years old. (a) SCH: there were 16 studies involving 21535 subjects with an average age younger than 45 years old. The result of the heterogeneity test was *I*^2^ = 97%, *P* < 0.01, *Z* = 4.84, *P* < 0.01, SMD = 0.95, 95% CI: 0.57–1.34, and the difference was statistically significant. 35 studies involved 12957 subjects with an average age between 45 and 60 years old. The result of the heterogeneity test was *I*^2^ = 95%, *P* < 0.01, *Z* = 6.67, *P* < 0.01, SMD = 0.73, 95% CI: 0.52–0.95, and the difference was statistically significant. 17 studies involved 4957 subjects with an average older than 60 years old. The result of the heterogeneity test was *I*^2^ = 88%, *P* < 0.01, *Z* = 3.71, *P* < 0.01, SMD = 0.38, 95% CI: 0.18–0.58, and the difference was statistically significant. It was suggested that regardless of age, UA levels in patients with SCH were higher than those with normal thyroid function (Figure 3(c)). (b) SCHyper: there were 4 studies involving 18735 subjects with an average age younger than 45 years old, 2 studies involving 390 subjects with an average age between 45 and 60 years old, and 1 study involving 249 subjects with an average older than 60 years old. There were no significant differences among the ages in the levels of UA in SCHyper patients (Figure 4(c)).
3. Comorbidities: of the 68 studies which compared levels of UA between patients with SCH and normal thyroid function subjects, only two involved a comparison of serum UA levels between chronic kidney disease patients with and without SCH, six involved cardiovascular diseases including coronary heart diseases and hypertension, eighteen pertained to metabolic syndromes (diabetes and dyslipidemia), four were focusing on severe preeclampsia, and the other three were on pregnancy. Furthermore, 35 studies involved a comparison of SCH-only patients and normal subjects. Serum UA levels were significantly higher in SCH patients combined with metabolic syndrome or severe preeclampsia or pregnancy than those without SCH. No difference in the levels of serum UA was found between chronic kidney disease and cardiovascular diseases patients with or without SCH. The results are shown in Figure 3(d). Of the 7 studies which compared levels of UA between patients with SCHyper and normal thyroid function subjects, only one involved a comparison of serum UA levels between chronic kidney disease patients with and without SCHyper. The serum UA levels were significantly higher in patients with SCHyper with chronic kidney disease than in those with chronic kidney disease but without SCHyper. There was no statistically significant difference in the levels of serum UA between patients with or without SCHyper in six studies that included patients with SCHyper alone (Figure 4(d)).

#### 3.2.5. Relationship between SCH and Serum UA Levels before and after Treatment

**Figure 5:**
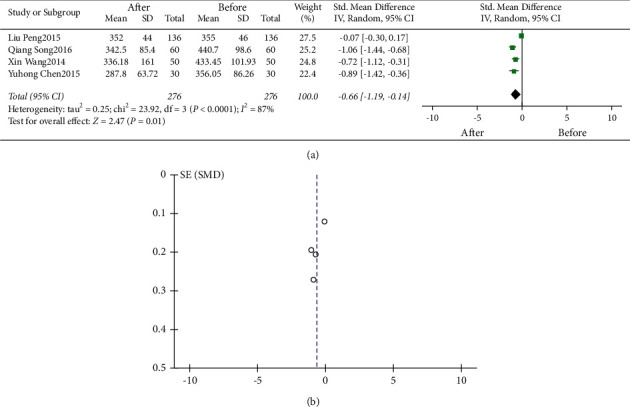
Relationship between SCH and serum UA levels before and after treatment. (a) Forest plot. (b) Funnel plot.

Four studies examined the level of UA in patients with SCH before and after treatment. The result showed that the level of UA reduced after treatment compared with before treatment (*I*^2^ = 87%, *P* < 0.01; *Z* = 2.47, *P* < 0.05, SMD = −0.66, 95% CI: −1.19 to −0.14, Figure 5(a)). The difference was statistically significant.

#### 3.2.6. Publication Bias

The funnel plot is shown in Figures 2(c), 2(d), 3(e), 4(e), and 5(b). Egger's test (*P* > 0.05) suggested no obvious publication bias.

## 4. Discussion

The results of this analysis showed that hyperuricemia was more prevalent in subclinical thyroid dysfunction than in normal thyroid function subjects. The serum UA levels of patients with SCH were significantly higher than that of patients with SCHyper and were higher than that of normal thyroid function subjects, with the difference being statistically significant.

Thyroid diseases include both hypo and hyperthyroidism with types of overt and subclinical [30].The relationship between overt thyroid dysfunction (hyperthyroidism and hypothyroidism) and UA has received considerable attention. Giordano et al. reported the prevalence of hyperuricemia was significantly higher among patients with hyperthyroidism and hypothyroidism compared with the general population [31]. Ford HC et al. also indicated that hyperthyroidism can cause hyperuricemia by increasing UA production or decreasing renal excretion [32]. The association between hypothyroidism and hyperuricemia was first proposed by Kuzell et al. in 1955 [33]; subsequent studies confirmed this association. A previous study showed that hyperthyroidism resulted in elevated levels of UA, but the increase was less than in hypothyroidism [31], which were similar to our results.

UA is the end product of the endogenous and dietary purine metabolism, which may be influenced by the thyroid hormones. It was reported that increased levels of UA were associated with reduced glomerular filtration rate (GFR) and renal plasma flow in hypothyroidism patients [25, 26, 34–38]. Furthermore, SCH could reduce cardiac contractility, and the GFR can decrease by 20–30% to below normal levels, thereby, changing reabsorption and secretion in the tubular, which simultaneously increases the level of uric acid, which results in a decrease in UA excretion [28]. The study by Desideri G et al. suggested that serum UA levels were significantly lower after replacement therapy with LT-4 in patients with iatrogenic SCH who had undergone thyroidectomy. Furthermore, it seemed that changes of UA levels are directly associated with changes of HOMA-IR. These observations further suggested that the effect of the UA metabolism in patients with recent-onset SCH was mediated by insulin sensitivity [27]. Several reports suggested that hyperuricemia could occur among hyperthyroidism participants. This could be due to the acceleration of the purine nucleotide metabolism during UA production [31, 39, 40].

In conclusion, this study provided a systemic analysis of the association between subclinical thyroid dysfunctions. Moreover, as most of the included studies are from China, our results may be more applicable to Chinese subjects. A limitation of the study is the retrospective data collection. One of the limitations of our study is the lack of a brief description of the purine content of foods with known effects on thyroid function. Inclusion and exclusion criteria for included studies are given in Table S2 in Supplementary Materials. Therefore, prospective longitudinal studies are needed to further confirm these results.

## 5. Conclusion

Subclinical thyroid dysfunction was associated with the higher prevalence of hyperuricemia. The levels of serum UA had significantly increased in SCH compared to SCHyper patients or normal controls. The level of UA decreased after treatment in patients with SCH.

## Data Availability

The data used to support this study are included within this article.

## Supplementary Materials

Supplementary Materials Table S1. Basic characteristics of included studies. Table S2. Inclusion and exclusion criteria and TSH level in patients with SCH/SCHyper of included studies.
