# Supplementary material for: The Association with Subclinical Thyroid Dysfunction and Uric Acid
Source: Int J Endocrinol. 2021 Dec 13;2021:9720618. doi: 10.1155/2021/9720618 (PMC8687809; doi:10.1155/2021/9720618)
Supplement: Supplementary Materials — Table S1. Basic characteristics of included studies. Table S2. Inclusion and exclusion criteria and TSH level in patients with SCH/SCHyper of included studies. [file 9720618.f1.zip › 9720618.f1/Table S1 (1).docx]

|  |  | **SCH** | | **euthyroid** | |  |  |  |  |
| --- | --- | --- | --- | --- | --- | --- | --- | --- | --- |
| **First Author** | **Year** | **Mean±SD** | **n** | **Mean±SD** | **n** |  | **Study type** | **Country** | **Reference** |
| Abdel-Gayoum A A | 2014 | 225.98±12.28 | 21 | 209.04±20.37 | 14 | mmol/l | Cross-sectional study | Saudi Arabia | Abdel-Gayoum AA.(2014) Dyslipidemia and serum mineral profiles in patients with thyroid disorders. Saudi Med J 35:1469-1476. |
| Afsar B | 2017 | 7.4±1.2 | 66 | 5.9±1.9 | 163 | mg/dl | Cross-sectional study | Turkey | Afsar B, Yilmaz MI, Siriopol D, Unal HU, Saglam M, et al.(2017) Thyroid function and cardiovascular events in chronic kidney disease patients. J Nephrol Res 30:235-242. |
| Ao Liu | 2018 | 311.75±74.84 | 66 | 300.2±74.18 | 67 | NA | Cross-sectional study | China | Liu A.(2018) Correlation between carotid atherosclerosis and urinary microalbumin in elderly patients with hypertension complicated with subclinical hypothyroidism.Master. China Medical University.(In Chinese) |
| Chuang Zhang | 2019 | 275.94±35.30 | 200 | 220.2±4.6 | 200 | NA | Case control study | China | Zhang Chuang.(2019) Study on the relationship between thyrotropin and cerebrovascular disease risk factors in subclinical hypothyroidism patients.Chinese Community Doctors 35:78+80.(In Chinese) |
| Chunjiang Yang | 2011 | 312.81±61.03 | 45 | 268.47±48.65 | 50 | umol/l | Case control study | China | Yang C J, Tu Q F, Yu L T.(2011) The effect of arteriosclerosis related indicators on subclinical hypothyroidism.Chinese Journal of Laboratory Diagnosis 15:2046-2048.(In Chinese) |
| Chunrong Wang | 2019 | 357.83±125.19 | 74 | 288.97±113.28 | 70 | mmol/l | Case control study | China | Wang C R.(2019)Analysis of changes in blood lipid, blood glucose and blood uric acid in patients with subclinical hypothyroidism.Clinical Laboratory Journal(Electronic Edition) 8:4-5.(In Chinese) |
| Cong Chen | 2017 | 371±95.4 | 102 | 312±91.4 | 770 | umol/l | Cross-sectional study | China | Chen C, Ren A, Yang J, Jing C, Dou P.(2017) Effects of subclinical hypothyroidism on serum uric acid levels in patients with type 2 diabetes mellitus. Chinese Journal of Clinical Healthcare 20:357-360.(In Chinese) |
| Daijiajia Yin | 2015 | 242.65±38.47 | 64 | 220.61±21.26 | 100 | NA | Case control study | China | Yin Daijiajia, Tan Yang, Zhang Yangao, Zhu Chunhua, Jin Xin,et.al.(2015) A clinical study of subclinical hypothyroidism on the related factors of cardiovascular disease.Chongqing Medicine 44:4227-4228+4231.(In Chinese) |
| Devika Tayal | 2009 | 5.93±4.95 | 98 | 5.87±2.53 | 198 | mg/dl | Cross-sectional study | India | Tayal DD, Chawla DR, Arora DS, Gupta DVK, Sohi MJS,et.al.(2009)DYNAMIC CHANGES IN BIOCHEMICAL MARKERS OF RENAL FUNCTIONS WITH THYROID STATUS - A STUDY IN INDIAN POPULATION. Internet Journal of Medical Update 4. |
| Fei Li | 2014 | 316.82±73.42 | 118 | 262.3±73.42 | 298 | umol/l | Cross-sectional study | China | Li Fei, Li Zhihong, Guo Shuqin, Zhao Lianchen, Lu Ruiqi, et.al.(2014)The effect of subclinical hypothyroidism on metabolic syndrome and intracranial atherosclerosis in the elderly.Chinese Journal of Arteriosclerosis 22:1118-1122.(In Chinese) |
| Gao F | 2017 | 277.9±78.07 | 95 | 229.94±41.46 | 54 | umol/l | Case control study | China | Gao F, Wang G, Xu J.(2017) Alteration of Hemostatic Parameters in Patients with Different Levels of Subclinical Hypothyroidism and the Effect of L-thyroxine Treatment. Ann Clin Lab Sci 47:29-35. |
| Guoqing Chen | 2013 | 242.27±37.75 | 50 | 199.54±21.26 | 60 | NA | Case control study | China | Chen Guoqing, Xu Yingjie, Xie Jiang, He Jun, et.al.(2013)Analysis of serum lipid, blood glucose and uric acid levels in patients with subclinical hypothyroidism.China Practical Medicine 8:121-122.(In Chinese) |
| Hua Zhong | 2014 | 380.1±82.6 | 63 | 341.5±81.6 | 418 | umol/l | Cross-sectional study | China | Zhong Hua, Chen Gang, Xie Haiyan, Yan Xiaowei, Qin Mingwei.(2014)Correlation between subclinical hypothyroidism and metabolic factors in elderly patients.Chinese Journal of Health Care and Medicine16:367-369.(In Chinese) |
| Jiadan Wang | 2016 | 333±86 | 202 | 317±79 | 202 | umol/l | Case control study | China | Wang Jiadan, Zhang Qiao, Shi Lixin, Peng Nianchun, Xu Shujing, et.al.(2016)Changes of serum 25 hydroxyvitamin D levels and cardiovascular risk factors in residents with subclinical hypothyroidism.Chinese General Practice 19:2671-2675.(In Chinese) |
| Jianting Zhong | 2009 | 398.2±136.26 | 80 | 289.6±118.21 | 78 | mmol/l | Case control study | China | Jianting Zhong, Xuan Wang, Ling Zhao, Chunyu Wang, Ling Fang, *et.al*.(2009) Changes in lipid, glucose and uric acid metabolism in subclinical hypothyroidism.*Chinese Journal of Misdiagnostics* **9**:7367-7368.(In Chinese) |
| Jiaren Zhou | 2014 | 411±92 | 46 | 378±114 | 109 | umol/l | Case control study | China | Zhou Jia-ren, Li Wei, Du Jun, Qiao Chong, Tang Tao, et.al.(2014)Correlation between changes in thyroid hormone levels and renal function in pregnant women with severe preeclampsia complicated with hypothyroidism.Chinese Journal of Obstetrics and Gynecology 49:811-815.(In Chinese) |
| Jingli Cheng | 2019 | 291.21±79.21 | 62 | 290.31±73.23 | 198 | umol/l | Cross-sectional study | China | Cheng Jingli, Zheng Miaoyan, Shan Chunyan, Yang Yanhui, Yang Juhong, et.al.(2019) The effect of subclinical hypothyroidism on the progression of early renal disease in type 2 diabetes mellitus. Chinese Journal of Diabetes2019:653-657.(In Chinese) |
| Jingyuan Ren | 2012 | 357.24±96.08 | 35 | 379.38±97.72 | 198 | umol/l | Cross-sectional study | China | Ren JY, Yu ZQ, Zhang YY, Wang WW, Yang M, et.al.(2012) Effects of subclinical hypothyroidism on dynamic blood pressure in elderly patients with hypertension.Journal of Chinese Practical Diagnosis and Therapy 26:534-536.(In Chinese) |
| Juanjuan Sun | 2018 | 361.12±107.2 | 25 | 368.69±111.99 | 86 | umol/l | Case control study | China | Sun Juanjuan. (2018)Clinical hypothyroidism in patients with chronic kidney disease in Central Asia.Master. Qingdao University.(In Chinese) |
| Jue Wang | 2014 | 366±123.45 | 47 | 286.96±95.51 | 45 | umol/l | Cross-sectional study | China | Wang Jue.(2014) Study on the correlation between subclinical hypothyroidism and renal function in elderly men.Master. Kunming Medical University.(In Chinese) |
| Junzheng Chen | 2012 | 349.8±127.56 | 54 | 279.9±115.73 | 48 | umol/l | Case control study | China | Chen Junzheng.(2012) Analysis of serum lipid and uric acid levels in patients with subclinical hypothyroidism. Journal of Clinical and Experimental Medicine 11:58-59.(In Chinese) |
| Krysiak R | 2014 | 405±50 | 16 | 345±52 | 18 | umol/l | Cross-sectional study | Poland | Krysiak R, Gilowski W, Szkrobka W, Okopien B.(2014)Different effects of fenofibrate on metabolic and cardiovascular risk factors in mixed dyslipidemic women with normal thyroid function and subclinical hypothyroidism. Cardiovascular therapeutics 32:264-269. |
| Leyin Xia | 2015 | 351.58±83.3 | 355 | 286.51±103.47 | 70 | umol/l | Case control study | China | Xia Leyin.(2015) Changes in serum uric acid levels before and after treatment of primary hypothyroidism and its significance. Henan Medical Research 24:57-58.(In Chinese) |
| Libo Liang | 2013 | 327.06±86.7 | 356 | 302.86±74.1 | 331 | umol/l | Case control study | China | Liang Libo, Zhang Mei, Huang Hengjian, Wang Youjuan, Li Shuangqing.(2013) Analysis of serum lipid, blood glucose and uric acid levels in clinical hypothyroidism patients from Central Asia. Journal of Sichuan University (Medical Sciences) 44:954-956.(In Chinese) |
| Liping Xiao | 2018 | 335.35±91 | 114 | 310.12±88.69 | 114 | umol/l | Case control study | China | Xiao Liping.(2018) Correlation analysis of subclinical hypothyroidism with vascular complications and TCM syndromes in type 2 diabetes mellitus.Master. Liaoning University of Traditional Chinese Medicine.(In Chinese) |
| Man Jin | 2016 | 334.3±98.18 | 95 | 357.97±84.53 | 153 | NA | Cross-sectional study | China | Jin Man.(2016) The relationship between subclinical hypothyroidism and coronary artery disease.Master. Jilin University.(In Chinese) |
| Min Guo | 2015 | 328.6±77.02 | 54 | 266.11±70.54 | 150 | mmol/l | Cross-sectional study | China | Guo Min, Fu-Qiang, Li Yi, Yang Yuan-Xing.(2015)Effects of subclinical hypothyroidism on metabolic risk factors in type 2 diabetes mellitus. Chinese Journal of Postgraduates of Medicine 2015:93-96.(In Chinese) |
| Mingling Deng | 2016 | 297.68±30.54 | 39 | 257.44±27.25 | 85 | umol/l | Cross-sectional study | China | Deng ML, Zhang QL.(2016) Correlation between intracranial atherosclerosis and subclinical hypothyroidism in elderly patients with type 2 diabetes mellitus. Journal of Xinjiang Medical University 39:193-195.(In Chinese) |
| Mustafa Altay | 2017 | 4.3±1 | 35 | 4.2±0.9 | 30 | mg/dl | Case control study | Turkey | Altay M, Karakoç MA, Çakır N, Demirtaş CY, Cerit ET, et.al.(2017) Serum Total Sialic Acid Level is Elevated in Hypothyroid Patients as an Atherosclerotic Risk Factor. Journal of Clinical Laboratory Analysis 31. |
| Ping Dou | 2016 | 306.19±87.76 | 52 | 297.18±90.65 | 948 | umol/l | Cross-sectional study | China | Dou Ping.(2016) Analysis of the relationship between subclinical hypothyroidism and nonalcoholic fatty liver disease in type 2 diabetes mellitus and related factors.Master. Anhui Medical University.(In Chinese) |
| Qian Jiang | 2020 | 302.2±75.34 | 25 | 309.81±91.63 | 299 | umol/l | Case control study | China | Jiang Q.(2020) Analysis of thyroid function and biochemical indices in type 2 diabetes mellitus complicated with Hashimoto's thyroiditis.Master. Lanzhou University.(In Chinese) |
| Qiang Song | 2016 | 440.7±98.6 | 60 | 438.1±105.2 | 60 | mmol/l | Case control study | China | Song Q.(2016) Effect of thyroxine on blood lipid, blood glucose and uric acid levels in elderly patients with subclinical hypothyroidism. The Journal of Medical Theory and Practice 29:1468-1470.(In Chinese) |
| Qiannan Du | 2018 | 289±92 | 55 | 311±107 | 287 | umol/l | Cross-sectional study | China | Du Qiannan.(2018) Study on the correlation between different thyroid function and carotid plaque in type 2 diabetic patients.Master. Ningxia Medical University.(In Chinese) |
| Qing Chen | 2010 | 298.2±90.03 | 119 | 230.07±99.26 | 79 | umol/l | Case control study | China | Chen Qing.(2010) Clinical study on the correlation between thyroid function and blood lipid.Doctor. Shandong University.(In Chinese) |
| Rong Huang | 2013 | 390.33±77.88 | 392 | 340.26±56.12 | 18167 | umol/l | Cross-sectional study | China | Huang R, Cao Q, Gu JL, Ma JH, Gu WW,et.al.(2013) Clinical study of subclinical thyroid dysfunction and hyperuricemia. Journal of Shanghai Jiaotong University(Medical Science) 2013, 33:1348-1355. |
| Rongrong Zhang | 2013 | 369.72±91.33 | 45 | 380.67±94.97 | 58 | umol/l | Case control study | China | Zhang Rong-rong, Li Wen-chao, Wang Zhe, Li Ming-long.(2013)Clinical observation on the effect of primary hypothyroidism on uric acid. Military Medical Sciences 37:836-838.(In Chinese) |
| Ruoxi Tang | 2016 | 329±78 | 73 | 309±90 | 371 | umol/l | Cross-sectional study | China | Tang RX, Fan Y.(2016) The status and influencing factors of subclinical hypothyroidism in type 2 diabetes mellitus patients in Xinjiang. Chinese General Practice 19:3452-3456.(In Chinese) |
| Saini V | 2012 | 6.03±0.27 | 77 | 4.9±0.3 | 120 | NA | Cross-sectional study | India | Saini V, Yadav A, Arora MK, Arora S, Singh R, et.al.(2012)Correlation of creatinine with TSH levels in overt hypothyroidism - a requirement for monitoring of renal function in hypothyroid patients Clinical biochemistry 45:212-214. |
| Sayari Saba | 2018 | 4.24±1.2 | 56 | 3.94±1.01 | 51 | mg/dl | Cross-sectional study | India | Saba S, Ziba M, Zohre T.(2018)The relationship between subclinical hypothyroidism and serum levels of uric acid and creatinine in children aged 2-14 years. Ann Pediatr Endocrinol Metab23. |
| Shunyou Deng | 2008 | 358.5±138.48 | 60 | 289.4±116.11 | 56 | mmol/l | Case control study | China | Deng S Y, Chen G Y, Zhang T.(2008) Analysis of serum lipid, blood glucose and uric acid in patients with subclinical hypothyroidism. Modern Journal of Integrated Traditional Chinese and Western Medicine 2008:816-817+824.(In Chinese) |
| Song Bo | 2010 | 359.5±138.48 | 35 | 289.5±116.23 | 35 | umol/l | Case control study | China | Bo Song, Shi Yishan, Wang Xiaolei, Shi Feng, Ju Wei, et.al.(2010) Changes of blood lipid and uric acid in 35 patients with subclinical hypothyroidism. Chinese Journal of Gerontology 30:3771-3772.(In Chinese) |
| Tianfang Fu | 2016 | 408.58±87.44 | 45 | 385.41±97.29 | 50 | umol/l | Cross-sectional study | China | Fu TF, Wang R, Li B.(2016) Effects of thyroid hormone levels on renal function in pregnant women with severe preeclampsia complicated with hypothyroidism. Hebei Medical Journal 38:1342-1344.(In Chinese) |
| Torkian P | 2020 | 5.1±1.4 | 118 | 4.6±1.63 | 121 | mg/dl | Case control study | Iran | Torkian P, Mansournia MA, Mansournia N.(2020) Evaluation of biochemical markers of kidney function in patients with subclinical hypothyroidism in comparison with euthyroid people. Journal of family medicine and primary care 9:4234-4239. |
| Wei Liu | 2015 | 386±94 | 78 | 357±87 | 105 | mmol/l | Cross-sectional study | China | Liu W, Pan M, Wu XH.(2015) The effect of subclinical hypothyroidism on metabolic factors in elderly patients with coronary heart disease. Chinese Journal of Clinical Healthcare 18:354-356.(In Chinese) |
| Wei Wei | 2019 | 252.7±22.5 | 114 | 246.4±24.2 | 235 | umol/l | Cross-sectional study | China | Wei W, Xie XQ, Liu JQ.(2019)Influential factors of menopausal female diabetes mellitus complicated with subclinical hypothyroidism. Chinese Journal of Prevention and Control of Chronic Diseases 27:93-96+101.(In Chinese) |
| Weina Xu | 2015 | 308.75±81.71 | 107 | 277.13±74.12 | 1348 | umol/l | Cross-sectional study | China | Xu W N, Zhang X F, Hu Z H.(2015) Metabolic risk factors of subclinical hypothyroidism associated with fatty liver.China Modern Doctor 53:56-59.(In Chinese) |
| Wen Cao | 2018 | 294.71±10.7 | 45 | 225.66±6.7 | 50 | umol/l | Case control study | China | Cao W, Zheng RD, Fan YF, Chen GF, Lu L.(2018) Effects of hypothyroidism on serum uric acid and glycolipid metabolism. Medical Journal of West China 30:1467-1470.(In Chinese) |
| Wenhui He | 2016 | 390.23±18.82 | 115 | 320.61±17.63 | 110 | mmol/l | Case control study | China | He WH, Li XL, Wang XJ.(2016) The effect of subclinical hypothyroidism during pregnancy on glucose and lipid metabolism in gestational diabetes mellitus.Hebei Medical Journal 38:2289-2291.(In Chinese) |
| Wenjuan Jiang | 2016 | 377.2±101.3 | 100 | 333.2±90.1 | 100 | umol/l | Case control study | China | Jiang WJ.(2016) Diagnosis of subclinical thyroid dysfunction and its correlation with blood glucose, lipid and uric acid metabolism in vivo.Modern Medical Journal 44:1374-1377.(In Chinese) |
| Wenping Li | 2020 | 429.46±90.61 | 57 | 385.04±87.24 | 118 | umol/l | Cross-sectional study | China | Li Wen-ping, ZHAO Hai-yan.(2020) Effects of hypothyroidism on clinical outcomes in patients with severe preeclampsia. Practical Journal of Medicine & Pharmacy 37:893-896.(In Chinese) |
| Wenzhu Yu | 2015 | 386±80 | 35 | 261±68 | 35 | umg/l | Case control study | China | Yu WZ, Yuan D, Fan YX, Guan Y.(2015)Correlation analysis of blood lipid, homocysteine and serum uric acid in patients with subclinical hypothyroidism. Chinese Journal of Laboratory Diagnosis 19:1496-1497.(In Chinese) |
| Xiaolei Chen | 2018 | 370.85±118.62 | 154 | 335.88±91.93 | 183 | umol/l | Case control study | China | Chen XL, Jia Nong, Ke TY, Tian LY, Cai LP, et.al.(2018)Correlation between subclinical hypothyroidism and renal impairment in type 2 diabetes mellitus. Chinese Journal of Practical Internal Medicine 38:65-68. |
| Xiaoyan Guo | 2019 | 284.58±76.2 | 85 | 248.79±69.77 | 85 | mmol/l | Case control study | China | Guo Xiaoyan.(2019) Changes of serum uric acid, glucose and lipid metabolism and adipokines in patients with subclinical hypothyroidism and their significance.Master. Shanxi Medical University.(In Chinese) |
| Xuelian Jiang | 2017 | 375±81 | 50 | 269±72 | 55 | umol/l | Cross-sectional study | China | Jiang Xuelian, Wang CW, Wu Shulan, Zhu Defa.(2017) Effects of mild subclinical hypothyroidism on carotid intima thickness in type 2 diabetes mellitus. Anhui Medical and Pharmaceutical Journal 21:1613-1615.(In Chinese) |
| Xueqin Wang | 2019 | 326.31±37.92 | 51 | 287.82±32.65 | 51 | umol/l | Case control study | China | Wang XQ, Wang W.(2019) Effects of subclinical hypothyroidism on blood glucose, lipid and uric acid levels in patients with diabetes mellitus. Journal of Preventive Medicine of Chinese People's Liberation Army 37:47-48.(In Chinese) |
| Yahui Liu | 2019 | 338.41±70.13 | 112 | 312.34±78.81 | 112 | umol/l | Case control study | China | Liu YH.(2019)Correlation analysis of early diabetic nephropathy and TCM constitution in patients with type 2 diabetes mellitus complicated by SCH.Master. Liaoning University of Traditional Chinese Medicine.(In Chinese) |
| Yanbin Zhang | 2017 | 352.72±54.26 | 424 | 284.33±65.79 | 613 | umol/l | Cross-sectional study | China | Zhang YB, He WH, Wang JY, Li X.(2017) The effect of subclinical hypothyroidism on the pathogenesis and metabolic status of pregnancy diseases.Maternal and Child Health Care of China 32:2591-2593.(In Chinese) |
| Yanhua Xi | 2013 | 272.13±87.03 | 30 | 268.97±85.72 | 30 | mmmol/l | Case control study | China | Yanhua Xi .(2013)Nesfatin-1 levels in patients with type 2 diabetes mellitus with metabolic syndrome and subclinical hypothyroidism.Master. Nanchang University.(In Chinese) |
| Yating Zhang | 2012 | 242.27±37.75 | 50 | 199.54±21.26 | 60 | NA | Case control study | China | Zhang Yating.(2012) Changes of blood lipid, blood glucose and uric acid levels in patients with subclinical hypothyroidism. Journal of Clinical Medicine in Practice 16:100-101+104.(In Chinese) |
| Yin Li | 2016 | 356.56±80.69 | 155 | 275.16±90.95 | 250 | umol/l | Cross-sectional study | China | Li Y, Cui H R, Luo Y.(2016) The effect of subclinical hypothyroidism on metabolic syndrome and carotid atherosclerosis. South China Journal of Cardiovascular Diseases22:188-191+226.(In Chinese) |
| Yingchuan Liu | 2017 | 368.52±135.94 | 67 | 320.87±95.38 | 352 | umol/l | Cross-sectional study | China | Liu YC, Tao JL, Shu W, Zhang M.(2017) Clinical characteristics and outcome analysis of patients with acute ST-segment elevation myocardial infarction complicated with subclinical hypothyroidism. Journal of Logistics University of PAP(Medical Sciences) 26:479-484. |
| Yuan Guo | 2018 | 306.53±83.88 | 108 | 297.44±83.5 | 216 | umol/l | Cross-sectional study | China | Guo Y, Zhang G Y, Ma S Q.(2018) Study on the relationship between subclinical hypothyroidism and pregnancy outcome. Journal of Ningxia Medical University 40:593-596.(In Chinese) |
| Yuelei Wu | 2020 | 339.256±124.032 | 129 | 337.866±106.047 | 131 | umol/l | Cross-sectional study | China | Wu Yuelei, Liu Shuyi, Cao Ying, Tang Dan, Ye Xiaoping,et.al.(2020) The relationship between subclinical hypothyroidism and blood pressure variability in elderly patients with hypertension. Chinese Journal of Gerontology 40:1127-1131.(In Chinese) |
| Yuhong Chen | 2015 | 356.05±86.26 | 30 | 274.29±62.2 | 20 | mmol/l | Case control study | China | Chen Yuhong.(2015) Changes of serum uric acid in subclinical hypothyroidism patients before and after replacement therapy.Journal of Heze Medical College 27:28-29+34.(In Chinese) |
| Yuqin Yuan | 2016 | 367±65 | 42 | 270±60 | 42 | umol/l | Case control study | China | Yuan Yuqin.(2016) Clinical analysis of serum lipids, C-reactive protein and uric acid in patients with subclinical hypothyroidism. Contemporary Medicine 22:50-51.(In Chinese) |
| Zhang J | 2016 | 304.09±87.62 | 710 | 322.32±87.58 | 3187 | NA | Cross-sectional study | China | Zhang J, Meng Z, Zhang Q, Liu L, Song K,et.al.(2016) Gender impact on the correlations between subclinical thyroid dysfunction and hyperuricemia in Chinese. Clinical rheumatology 35:143-149. |
| Zhangxia Cui | 2016 | 447.9±87.5 | 42 | 391.8±90.5 | 32 | umol/l | Cross-sectional study | China | Cui Zhangxia.(2016) Correlation between thyroid hormone levels and renal function in patients with severe preeclampsia complicated with hypothyroidism. Chinese Journal of Family Planning & Gynecotokology 8:29-31. |
| Zhiling Hao | 2012 | 389.2±89.41 | 31 | 323.71±64.71 | 40 | mmol/l | Case control study | China | Hao Zhiling.(2012) Clinical analysis of changes in serum serum and renal uric acid and hs-CRP levels in patients with thyroid dysfunction. Guide of China Medicine 10:259-261.(In Chinese) |
|  |  | **SH** | | **euthyroid** | |  |  |  |  |
| **First Author** | **Year** | **Mean±SD** | **n** | **Mean±SD** | **n** |  |  |  |  |
| Abdel-Gayoum A A | 2014 | 213.54±17.29 | 24 | 209.04±20.37 | 14 | mmol/l | Cross-sectional study | Saudi Arabia | Abdel-Gayoum AA.(2014) Dyslipidemia and serum mineral profiles in patients with thyroid disorders. Saudi Med J 35:1469-1476. |
| Qing Chen | 2010 | 264.67±92.98 | 119 | 230.07±99.26 | 79 | umol/l | Case control study | China | Chen Qing.(2010) Clinical study on the correlation between thyroid function and blood lipid.Doctor. Shandong University.(In Chinese) |
| Rong Huang | 2013 | 267.13±71.54 | 172 | 340.26±56.12 | 18167 | umol/l | Cross-sectional study | China | Huang R, Cao Q, Gu JL, Ma JH, Gu WW,et.al.(2013) Clinical study of subclinical thyroid dysfunction and hyperuricemia. Journal of Shanghai Jiaotong University(Medical Science) 2013, 33:1348-1355. |
| Wen Cao | 2014 | 315.51±74.59 | 50 | 292.07±62.32 | 110 | umol/l | Case control study | China | Cao W, Zheng RD, Fan YF, Chen GF, Lu L.(2018) Effects of hypothyroidism on serum uric acid and glycolipid metabolism. Medical Journal of West China 30:1467-1470.(In Chinese) |
| Wenjuan Jiang | 2016 | 304.2±73.7 | 100 | 333.2±90.1 | 100 | umol/l | Case control study | China | Jiang WJ.(2016) Diagnosis of subclinical thyroid dysfunction and its correlation with blood glucose, lipid and uric acid metabolism in vivo.Modern Medical Journal 44:1374-1377.(In Chinese) |
| Zhengxia Di | 2019 | 308.04±84.01 | 49 | 280.93±95.74 | 200 | umol/l | Cross-sectional study | China | Di Zhengxia, Tian Wenjun, Shao Junfeng, Sun Xiaolin, Zhang Yun.(2019)Investigation of thyroid dysfunction in physical examination population. Laboratory Medicine and Clinic 16:1172-1175.(In Chinese) |
| Afsar B | 2017 | 6.8±1.3 | 27 | 5.9±1.9 | 163 | mg/dl | Cross-sectional study | Turkey | Afsar B, Yilmaz MI, Siriopol D, Unal HU, Saglam M, et al.(2017) Thyroid function and cardiovascular events in chronic kidney disease patients. J Nephrol Res 30:235-242. |
| **SCH** |  | **Before treatment** | | **After treatment** | |  |  |  |  |
| **First Author** | **Year** | **Mean±SD** | **n** | **Mean±SD** | **n** |  |  |  |  |
| Liu Peng | 2015 | 352±44 | 136 | 355±46 | 136 | NA | Cross-sectional study | China | Peng L, Ruidong L, Xia C, Yingying C, Debao W, et.al.(2015)Can levothyroxine treatment reduce urinary albumin excretion rate in patients with early type 2 diabetic nephropathy and subclinical hypothyroidism? A randomized double-blind and placebo-controlled study. Current medical research and opinion , 31.(In Chinese) |
| Qiang Song | 2016 | 342.5±85.4 | 60 | 440.7±98.6 | 60 | mmol/l | Case control study | China | Song Q.(2016) Effect of thyroxine on blood lipid, blood glucose and uric acid levels in elderly patients with subclinical hypothyroidism. The Journal of Medical Theory and Practice 29:1468-1470.(In Chinese) |
| Xin Wang | 2014 | 336.18±161 | 50 | 433.45±101.93 | 50 | mmol/l | Cross-sectional study | China | Wang X, Guo JH.(2014) Effects of thyroxine on serum lipid, blood glucose and uric acid levels in elderly patients with subclinical hypothyroidism.Chinese Journal of Gerontology 34:637-639.(In Chinese) |
| Yuhong Chen | 2015 | 287.8±63.72 | 30 | 356.05±86.26 | 30 | mmol/l | Case control study | China | Chen Yuhong.(2015) Changes of serum uric acid in subclinical hypothyroidism patients before and after replacement therapy.Journal of Heze Medical College 27:28-29+34.(In Chinese) |
|  |  | **SCH** | | **euthyroid** | |  |  |  |  |
| **First Author** | **Year** | **Hyperuricemia（n）** | n总 | **Hyperuricemia（n）** | **n总** |  |  |  |  |
| Mei-hsing | 2004 | 90 | 1700 | 1968 | 39267 |  | Cohort study | China | MD MhC, PhD KML, MPH YMH, MD PYPW, MPH YCC, et.al.(2016)Abnormal Thyroid‐Stimulating Hormone and Chronic Kidney Disease in Elderly Adults in Taipei City. J Am Geriatr Soc 64. |
| Quanyu Li | 2013 | 55 | 148 | 51 | 152 |  | Case control study | China | Quanyu Li,Xiulian Ren，Heng Su,Ben Niu，Yun Zhang et.al.(2013)Clinical and biochemical characteristics of patients with type 2 diabetes mellitus combined with subclinical hypothyroidism. Journal of Kunming Medical University.9:66-68. |
| Rong Huang | 2013 | 74 | 392 | 2284 | 18167 |  | Cross-sectional study | China | Huang R, Cao Q, Gu JL, Ma JH, Gu WW,et.al.(2013) Clinical study of subclinical thyroid dysfunction and hyperuricemia. Journal of Shanghai Jiaotong University(Medical Science) 2013, 33:1348-1355. |
|  |  | **SH** | | **euthyroid** | |  |  |  |  |
| **First Author** | **Year** | **Hyperuricemia（n）** | **n总** | **Hyperuricemia（n）** | **Total（n）** |  |  |  |  |
| Rong Huang | 2013 | 17 | 172 | 2284 | 18167 |  | Cross-sectional study | China | Huang R, Cao Q, Gu JL, Ma JH, Gu WW,et.al.(2013) Clinical study of subclinical thyroid dysfunction and hyperuricemia. Journal of Shanghai Jiaotong University(Medical Science) 2013, 33:1348-1355. |
| Zhang J | 2016 | 17 | 103 | 516 | 3187 |  | Cross-sectional study | China | Zhang J, Meng Z, Zhang Q, Liu L, Song K,et.al.(2016) Gender impact on the correlations between subclinical thyroid dysfunction and hyperuricemia in Chinese. Clinical rheumatology 35:143-149. |

Table S1 Basic characteristics of included studies
