# Supplementary material for: The Association with Subclinical Thyroid Dysfunction and Uric Acid
Source: Int J Endocrinol. 2021 Dec 13;2021:9720618. doi: 10.1155/2021/9720618 (PMC8687809; doi:10.1155/2021/9720618)
Supplement: Supplementary Materials — Table S1. Basic characteristics of included studies. Table S2. Inclusion and exclusion criteria and TSH level in patients with SCH/SCHyper of included studies. [file 9720618.f1.zip › 9720618.f1/Table S2.docx]

Table S2 Inclusion and exclusion criteria and TSH level in patients with SCH/SCHyper of included studies

| First Author | Year | diagnostic criteria | Inclusion and Exclusion criteria | Measuring method | TSH |
| --- | --- | --- | --- | --- | --- |
| Abdel-Gayoum A A | 2014 | SCH：TSH ≥7 mIU/ml and with normal FT3 and FT4  SCHyper ：Patients with TSH ≤0.1 mIU/ml and normal FT3 and FT4 | Inclusion criteria included all newly diagnosed patients, males and females aged 12-97 years, and hypothyroid patients receiving thyroxine replacement therapy.  Exclusion criteria included patients with abnormal liver function, chronic kidney disease, or overt diabetes mellitus. | FT3, FT4, TSH ：Autoanalyzer (ELecsys 2010, Cobas E 411, Mannheim Germany).  Uric acid ：Dimension RxL-Max, Germany. | NA |
| Afsar B | 2017 | SCHyper: TSH Level ＜0.35μIU/ml but fT3 and fT4 levels within reference ranges;  SCH: TSH level≥5.5μIU/ml but fT3 and fT4 levels within reference ranges; The reference values were: TSH: μIU/ml(0.35–5.5); fT3: pg/ml (2.3–4.2); fT4: ng/dl (0.89–1.76). | Exclusion criteria including acute infections and unwillingness to participate in the study were applied.Concomitant treatment with drugs interacting with thyroid function (amiodarone, thyrostatic agents, or corticosteroids equivalent to prednisone C10 mg/day), implausible thyroid test results, or incomplete thyroid tests | TSH, fT3 and fT4 ：the Modular Analytıcs E 170 Module (Roche Diagnostics, Indianapolis,USA). | 0.16μIU/ml（SCHyper）  8μIU/ml（SCH） |
| Ao Liu | 2018 | SCH：TSH>4.94mU/L，but fT3 and fT4 levels within reference ranges;（FT32.63~5.7，FT49.01~19.05） | All patients were required to exclude secondary hypertension, hypothyroidism, hyperthyroidism, postoperative thyroid, diabetes, severe renal insufficiency, persistent atrial fibrillation, severe heart rate disorder, acute myocardial infarction, heart failure, viral hepatitis and hematological diseases, various infectious diseases, rheumatic diseases, and malignant tumors. | NA | 7.13mIU/L |
| Chuang Zhang | 2019 | NA | Inclusion criteria: meet the clinical diagnostic criteria for hypothyroidism; first onset, no previous drug treatment; informed and agreed to participate in the study. Exclusion criteria: combined with severely impaired liver and kidney function; combined with diabetes, hypertension, and metabolic syndrome; with a history of familial hyperhomocysteinemia. | Uric acid: determination by fully automatic analyzer and supporting reagent.  TSH: measured by radioimmunoassay | 9.52mIU/L |
| Chunjiang Yang | 2011 | NA | Exclusion criteria: ① patients with pituitary hypothyroidism; (2) combined with tumor, liver and kidney disease, diabetes mellitus; ③ Patients with hereditary hyperlipidemia and hypertension; ④ Receive drug treatment that affects blood uric acid, blood lipid and thyroid stimulating hormone | Uric acid: measured by Hitachi 7600 automatic biochemical analyzer  FT3, FT4, TSH: using radioimmunoassay, the kit is provided by Beijing Furui Company  Provided, the normal reference range of TSH is 4.2 mU/L -6 .5 mU/L | 12.48mIU/L |
| Chunrong Wang | 2019 | SCH：TSH>4.2 Mu/L，but fT3 and fT4 levels within reference ranges | Inclusion criteria: all patients met the criteria of subclinical hypothyroidism (TSH > 4.2mu/l, and FT4 and FT3 were normal). They did not take drugs affecting the metabolism of blood lipid, blood glucose and blood uric acid three months before the study. They were informed of the study and signed the informed consent form.  Exclusion criteria: exclude patients with pituitary tumor, diabetes, malignancy, psychosis or family history of mental illness, and disagree to participate in the study. | Uric acid: detected by automatic biochemical analyzer and supporting reagents;  TSH, FT3, FT4: detected by radioimmunoassay. | 9.67mIU/L |
| Cong Chen | 2017 | ① SCH: TSH ＞ 4.34 mIU / L, FT3 and FT4 are in the normal range. ② HUA: under normal purine diet, fasting blood uric acid was detected twice on a different day, with male and postmenopausal women > 420 μ Mol / L, premenopausal women > 360 μ mol /L。 | All subjects met the diagnostic criteria of type 2 diabetes in 1999, and ruled out type 1 diabetes, late onset autoimmune diabetes, severe acute complications, severe heart, liver and kidney diseases, etc., resulting in low three iodine thyroxine syndrome, past thyroid disease, familial hyperlipidemia, malignant tumor, hematological disease, chronic kidney disease, and so on. All the subjects were diagnosed as type 1 diabetes mellitus. Diabetes insipidus and other internal and external diseases affecting the level of uric acid and blood lipid, as well as the use of diuretics, aspirin and other drugs affecting uric acid and blood lipid. | TSH, FT4, FT3: chemiluminescence enzyme linked immunosorbent assay. Uric acid: determination by automatic biochemical instrument | 6.2mIU/L |
| Daijiajia Yin | 2015 | SCH：ＴＳＨ＞ ４．８ｍＩＵ／Ｌ，FT3 and FT4 are in the normal range. | All patients were found for the first time and did not receive drug treatment. Discharge criteria: (1) patients with a history of familial hyperhomocysteinemia; (2) suffering from hypertension, diabetes, cancer and metabolic syndrome; (3) Severe impairment of liver and kidney function; (4) Suffering from other endocrine and metabolic diseases; (5) Those who used folic acid and vitamin B12 drugs in recent 3 months. | Uric acid: determined by automatic biochemical analyzer and supporting reagents.  TSH: measured by radioimmunoassay | 9.428mIU/L |
| Devika Tayal | 2009 | SCH： TSH- 6.1 to19.9μIU/ml with normal T4 and T3 levels  overt hypothyroid：TSH ≥ 20 μIU/ml and/ or abnormally low T4 and T3 levels. | Patients were clinically evaluated to rule out hypertension, diabetes mellitus or any other medical condition, which may affect the renal function | Serum TSH, T4 and T3 ： ELISA kits obtained from Ranbaxy, India. Serum T3 and T4 were performed using competitive ELISA technique and serum TSH was performed using sandwich ELISA technique. The normal ranges for TSH, T4 and T3 values were 0.39-6.16μIU/ml, 4.4-10.8μg/dl, and 0.52-1.85ng/ml respectively.  Serum uric acid levels were determined on Olympus AU 400 autoanalyser using standard kits.Uric acid by enzymatic end-point method. | 11.9mIU/L |
| Fei Li | 2014 | SCH : TSH ＞ 4. 20 mIU /L，FT3 and FT4 are in the normal range. | Exclude those who currently or previously have thyroid diseases, diseases that may cause abnormal thyroid hormone metabolism, and those who are or recently take drugs that may affect thyroid function; Pregnancy status. | Uric acid: the full-automatic biochemical analyzer and original kit of Beckman Kurt company in the United States are used for determination. | ≥4.2mIU/L |
| Gao F | 2017 | Thyroid stimulating hormone (TSH) levels >4.2 µIU/mL and serum free T4 (FT4) and free T3 (FT3) concentration within the normal laboratory range. | All patients were newly diagnosed SH and no previous or ongoing L-T4 therapy. Inclusion criteria included thyroid stimulating hormone (TSH) levels >4.2 µIU/mL and serum free T4 (FT4) and free T3 (FT3) concentration within the normal laboratory range.  Exclusion criteria were as follows: age below 18 years; inherited alterations in primary and/or secondary hemostasis; personal and/or family history of arterial or venous thrombosis;the taking of medications including anticoagulant or an-  tiplatelet drugs, drugs of affecting thyroid function, insulin, hypoglycemic drugs, corticosteroid, hypolipidemic drugs, oral contraceptive intake, or antihypertensive drugs; history of other disease known to impact hemostatic variables levels (liver disease, hepatitis, renal failure, coronary heart disease, malignancy, hematological diseases, active inflammatory processes, pregnancy, puerperium); and missing data. | TSH,FT3, FT4, ：full-automatic immune analyzer by electro-chemiluminescence method (cobas e601, Roche, Berlin,Germany), with normal reference ranges of 0.27–4.2 µIU/mL, 2–4.4 pg/mL, 0.93–1.7 ng/dL, <115 IU/mL, and<34 IU/mL, respectively.  UA ： full-automatic biochemistry  analyzer (7600-120 HITACHI, Japan), with normal reference ranges of 3.6-6.1mmol/L | 9.18mIU/L |
| Guoqing Chen | 2013 | SCH：TSH ＞ 10 mIU /L，FT3 and FT4 are in the normal range. | No drug treatment and no other serious endocrine diseases | Uric acid: automatic biochemical analyzer (hd-f2600. Hanfang Medical Instrument Co., Ltd.) | ≥10mIU/L |
| Hua Zhong | 2014 | SCH: TSH increased and FT4 was in the normal range; Hyperuricemia: male UA > 420 μ Mol / L, female UA > 357 μ mol/L.Normal range of thyroid hormone levels：TSH 0.38 ～ 4.34 mIU/L，T3 0.66 ～ 1.92 ng/ml，FT3 1.8 ～4.1 pg/ml，T4 4.30 ～ 12.50 μg/dl，FT4 0.81 ～ 1.89ng/dl. | Exclusion criteria: (1) definite diagnosis of thyroid dysfunction and taking thyroid hormone replacement therapy or antithyroid therapy; (2) History of thyroid surgery; (3) Chronic cardiac insufficiency, below NYHA class II; (4) Severe liver dysfunction; (5) Chronic kidney disease (CKD) stage 3 and below; (6) Malignant tumor; (7) Recent acute infection; (8) There was a history of trauma or surgery within 6 months. | NA | ≥4.34mIU/L |
| Jiadan Wang | 2016 | Normal range of thyroid hormone levels: TSH 0. 71 ～ 6. 25 mU / L，FT3 3. 1 ～ 6. 8 pmol / L，FT4 12. 0 ～ 22. 0 pmol / L，TPOAb ≤ 34 U /ml，TGAb ≤ 115U /ml.  SCH: TSH ＞ 6. 25 mU / L，FT3 and FT4 are in the normal range. | Inclusion criteria: (1) the subclinical hypothyroidism group met the diagnostic criteria of subclinical hypothyroidism [9]; (2) The control group met the diagnostic criteria of normal thyroid function and non thyroid antibody positive [9]. Exclusion criteria: digestive system diseases, abnormal liver and kidney function, taking hormone drugs. | TSH, FT3 and FT4: detected by electrochemiluminescence (cobas-e601 automatic immunoluminescence analyzer of Roche) | 8.1mIU/L |
| Jianting Zhong | 2009 | SCH：TSH > 8 mu / L (normal reference range 0.2 ~ 8.0 mu / L), FT4 is normal (normal reference range 9.0 ~ 23.5 PMO L / L), and the patient has no obvious hypothyroidism symptoms and signs. | All patients were found for the first time and were not treated. All subjects did not have familial hyperlipidemia, hypertension, diabetes, liver disease, and kidney disease. They did not take drugs that affect sugar, fat, protein, blood pressure and uric acid metabolism in the past 3 months. | UA: determined by automatic biochemical analyzer and supporting reagent. TSH, FT3 and FT4 were determined by radioimmunoassay. | 13.62mIU/L |
| Jiaren Zhou | 2014 | Normal range of thyroid hormone levels：  TSH 0．36～3．30 mU／L，FT4:9．21～16．50  pmol／L，FT3:2．92～5．70 pmol／L.Clinical hypothyroidism in the second and third trimester of pregnancy: TSH level > 3.3 mu / L, FT4 < 9.21 pmol / L; SCH: TSH > 3.3 mu / L, FT4 and FT3 levels were normal. | Previous history of thyroid disease, nephropathy, diabetes and chronic hypertension were excluded. All were singleton pregnancies. Thyroid diseases: those with positive thyroid peroxidase antibody (TPO AB) and thyroglobulin antibody (TG AB) were excluded; Those with personal and family history of thyroid disease were excluded; Exclude those with visible or palpable goiter; Those taking drugs (except estrogen) were excluded. | TSH, FT3 and FT4 were determined by chemiluminescent particle immunoassay using Abbott architect 2000 kit produced by Abbott Corporation of the United States;  Uric acid: determined by enzyme colorimetry with Roche DDP automatic biochemical analyzer | 6.1mIU/L |
| Jingli Cheng | 2019 | SCH: TSH > 4.2mIU/L, FT4 and FT3 levels were normal. | Inclusion criteria: (1) age 18 ~ 75 years; (2) meet the diagnostic criteria of WHO diabetes in 1999. (3) 24h urinary microalbumin (24h ‐ UMA) was 30 ~ 300mg / 24h during the first hospitalization; (4) EGFR is greater than 60ml · min ‐ 1 · (1.73m2) - 1 at the first hospitalization; (5) Patients with normal thyroid function (serum TSH 0.27 ~ 4.20mu/l, FT30.35 ~ 5.00mu/l, FT49.5 ~ 24.5pmol/l are normal) or meeting the diagnostic criteria of Sch (serum TSH > 4.2mu/l, FT3 and FT4 are within the normal range) and without thyroid hormone replacement therapy. Exclusion criteria: (1) acute metabolic disorders, such as diabetic ketoacidosis, hyperglycemia and hypertonic state, and acute severe infection. (2) Complicated with primary glomerulonephritis or other urinary system diseases; (3) Pregnancy, central hypothyroidism, Cushing's syndrome and other endocrine diseases; (4) Patients with previous thyroid history or thyroid drug treatment, and those who have taken phenytoin sodium, iodine, amiodarone and other drugs that affect thyroid function in recent 3 months; (5) Serious heart, liver and brain diseases and malignant tumors. | Uric acid: AU5400 automatic biochemical equipment produced by beckmancu company of the United States is adopted.  TSH, FT3, FT4: determination by Electrochemiluminescence | 6.72mIU/L |
| Jingyuan Ren | 2012 | SCH: TSH increased and FT4，FT3 was in the normal range | Inclusion criteria: all patients were ≥ 60 years old, had not taken antihypertensive drugs or had previously taken long-acting drugs and had stopped taking them for more than 2 weeks; Systolic blood pressure (SBP) ≥ 140mmHg (1mmhg = 0.133kpa) and / or diastolic blood pressure (DBP) ≥ 90mmHg. Exclusion criteria: age < 60 years old or being treated with antihypertensive drugs; Secondary hypertension, diabetes, heart valve disease, stroke, transient ischemic attack, coronary heart disease, heart failure and heart failure symptoms, but left ventricular ejection fraction < 50% and liver and renal insufficiency. The replacement dose of levothyroxine in patients with hyperthyroidism, hypothyroidism and hypothyroidism is insufficient. Patients in the recovery period of severe diseases temporarily increase thyroid stimulating hormone, patients in the recovery period of destructive thyroiditis, untreated primary adrenocortical insufficiency, patients injected with TSH and taking drugs affecting thyroid function | NA | 7.06mIU/L |
| Juanjuan Sun | 2018 | SCH: TSH > 4.2mIU/L, FT4 and FT3 levels were normal(FT3 3.1~6.8pmol/L；FT4 12~22pmol/L.TSH 0.27~4.2uIU/ml.) | The main causes of the disease were diabetic nephropathy, primary or secondary glomerulonephritis, hypertension and renal damage, and the age was greater than or equal to 18 years old. Exclusion criteria: 1. Patients with hemodialysis, peritoneal dialysis and previous history of thyroid disease; 2. Clinical hypothyroidism and hyperthyroidism; 3. Take drugs that affect thyroid function, such as glucocorticoid, amiodarone, antiepileptic drugs, tyrosine kinase inhibition, etc. Non CKD people who underwent routine physical examination in the health examination center of the hospital were used as the control group, except those with a previous history of thyroid disease. | FT3, FT4, TSH: Provided by Roche Diagnostics Shanghai Co., Ltd. Use electrochemiluminescence method to detect. | ≥4.2mIU/L |
| Jue Wang | 2014 | SCH: TSH increased and FT4，FT3 was in the normal range | Inclusion criteria: Elderly male patients aged 60 years or older with serum creatinine < 133umol / l have not taken drugs that affect blood lipid, blood uric acid and thyroid function recently. Exclusion criteria: hypertension and diabetes. The blood creatinine exceeds the upper limit of the normal reference range, and the upper limit of the normal range measured by the First Affiliated Hospital of Kunming Medical University is 133 μ mol/L； Stress and abnormal liver function; Autoimmune diseases; Urinary tract infection, primary renal disease; Recently take drugs that affect blood lipid, blood uric acid and thyroid function. | Uric acid: use OlympusAU2700 automatic biochemical analyzer to determine with enzymatic method and oxidase colorimetric method to determine | 8.69μIU/L |
| Junzheng Chen | 2012 | SCH: TSH increased and FT4，FT3 was in the normal range | All subjects had no malignancies, liver and kidney diseases, diabetes, hereditary hyperlipidemia, hypertension, and did not take drugs that affect blood sugar, blood lipids and blood uric acid metabolism in the past 3 months. | FT3, FT4, TSH: Measured by Japan Tosoh AIA-360 automatic fluorescent magnetic microparticle enzyme immunoassay analyzer  UA: The detection reagent is produced by Beijing Haomai Biological Engineering Co., Ltd., and it is measured by Hitachi 7 080 automatic biochemical analyzer | 10.46mIU/L |
| Krysiak R | 2014 | SCH：plasma TSH levels more than 4.5 mU/L but below 10 mU/L | The patients were eligible to participate in the study if they (1) were women between the ages of 30 and 65 years, (2) met the criteria of mixed dyslipidemia (total cholesterol and triglyceride levels more than 200 mg/dL and LDL cholesterol levels above 130 mg/dL), (3) complied with lifestyle modifications for at least 3 months preceding the study, (4) had free thyroid hormone levels within the reference range, (4) were medically stable, and, in the judgment of the investigators, (5) otherwise acceptable for entry on the basis of the findings of medical history, physical examination, and routine laboratory tests.  We excluded patients with any form of coronary artery disease, stroke within 6 months preceding the study, symptomatic congestive heart failure, diabetes, hyperprolactinemia, moderate or severe arterial hypertension (ESC/ESH grade 2 or 3), any acute and chronic inflammatory processes, autoimmune disorders, chronic pancreatitis, impaired renal or hepatic function, nephrotic syndrome, liver and biliary tract diseases, body mass index above 35 kg/m2 and poor patient compliance. No woman received other hypolipidemic drugs, drugs affecting plasma lipid levels or known to interact with fibrates within 3 months before the study. | Uric acid ：routine laboratory techniques (Roche Diagnostics, Basel, Switzerland; Instruments GmbH, Marburg, Germany).  TSH、FT4、FT3: electrochemiluminescence immunoassay method (Roche Diagnostics, Basel, Switzerland). | 7.8mIU/L |
| Leyin Xia | 2015 | NA | Exclusion criteria: Patients with a history of thyroid or pituitary surgery; Previous thyroid disease; Patients with hypoproteinemia and other systemic diseases that may affect thyroid function; Recent use of glucocorticoids, estrogen, antiepileptic drugs and other drugs affecting thyroid function. | FT3, FT4, TSH: Detected by Beckman DXI800 chemiluminescence immunoassay analyzer;  Uric acid: Detected by American Beck-man LX20 automatic biochemical analyzer | NA |
| Libo Liang | 2013 | SCH：ＴＳＨ＞４．２ｍＵ／Ｌ, FT4，FT3 was in the normal range | All patients were excluded from pituitary adenoma, diabetes, familial hyperlipidemia, liver disease, and malignant tumor. No drugs were found to affect blood sugar, blood lipid and blood uric acid metabolism in the past 3 months. | TSH, FT3, FT4: Roche E170 electrochemical luminometer is used, and the reagent is Roche's original set of reagents. | 5.53mIU/L |
| Liping Xiao | 2018 | SCH: TSH increased and FT4，FT3 was in the normal range(FT33.39-6.47pmol/L，FT：10.29-21.88pmo1l/L，TSH：0.3-3.6uIU/ml) | Exclusion criteria: (1) type 1 diabetes mellitus; (2) patients with acute diabetic complications; (3) Severe liver, kidney and heart dysfunction; (4) In recent 3 months, I have taken drugs that can affect nail function (such as amiodarone); (5) Thyroid diseases that have been clearly diagnosed and / or are being treated (such as hypothyroidism, thyroiditis, etc.); （6）TSH>10uIU/ml。 (7) Patients with malignant tumor; (8) Incomplete medical records | NA | 5.65mIU/L |
| Liu Peng | 2015 | SCH：TSH >4.0mIU/L, and FT3 and FT4 levels within the normal range | The inclusion criteria were as follows: patients that met the World Health Organization diagnostic criteria (1999) for T2DM, the Mogenson DN diagnosis and staging criteria,and the criteria for diagnosis  of Hashimoto thyroiditis and SCH.Age <65 years; Adequate glycemic control and a stable illness condition. no administration of angiotensin converting enzyme inhibitors, angiotensin receptor blockers,uric acid synthesis-inhibiting drugs, uricosuric drugs or lipid lowering drugs for nearly three months. The exclusion criteria were as follows: primary renal diseases; previous history of thyroid diseases and use of drugs affecting thyroid function; association with hypertension, severe dyslipidemia, acute metabolic disorders in diabetes, malignancy, or severe cardiac, hepatic, and cerebral diseases. The termination criteria were as follows: serum TSH was gradually increased more than 10.0mIU/L, progressed to clinical hypothyroidism, or decreased less than 4.0mIU/L in the observation period; serious adverse drug reaction that patients were unable to tolerate. | Uric acid : standard enzymatic procedures on an automated bioanalyzer (7600-020; Hitachi, Tokyo, Japan).  FT3, FT4, TSH: a radioimmunoassay (Linco Research, St. Charles, MO, USA). | 6.1mIU/L |
| Man Jin | 2016 | SCH: According to the management guidelines for subclinical hypothyroidism published by ETA in 2013, it is concluded that TSH is greater than the upper limit of the reference range, FT3 and FT4 are at normal levels, and there are no obvious symptoms and signs of hypothyroidism. | Inclusion criteria 1) age 45-80 years; 2) Thyroid hormone levels were measured; 3) All patients underwent coronary angiography. 4) Exclusion criteria for patients with cardiac function grade I ~ I Ⅱ 1) the statistical data are not complete; 2) Patients with primary hypothyroidism; 3) Patients with thyroxine replacement therapy who need to adjust the dosage due to their condition; 4) Severe liver and kidney dysfunction; 5) Hyperthyroidism and clinical hypothyroidism; 6) Pituitary tumor and malignant tumor; 7) Those taking iodine, amiodarone, metoclopramide, domperidone and other drugs in recent 3 months | NA | NA |
| Min Guo | 2015 | SCH：TSH>4．20 mU/L， and FT4，FT3 was in the normal | Exclusion criteria: previous history of thyroid diseases, central hypothyroidism and secondary hypothyroidism, recent use of drugs affecting thyroid function such as amiodarone; Acute complication of diabetes mellitus. Heart, liver, kidney diseases and malignant tumors. | Uric acid: using AU5421 automatic biochemical analyzer | 9.92mIU/L |
| Mingling Deng | 2016 | SCH:TSH＞4． 20 mU/L | Exclusion criteria: (1) patients with hematological diseases, tumors and liver and kidney diseases; (2) Previous or current diseases or thyroid diseases that may lead to hypothyroidism; (3) Recently or taking drugs that affect thyroid function; (4) Pregnant women; (5) Hyperuricemia and hyperlipidemia. | Thyroid function: use cobass411 automatic immunoassay analyzer | NA |
| Mustafa Altay | 2017 | Patients with elevated serum concentrations of TSH on at least two measurements and decreased fT4 concentrations were regarded as having overt hypothyroidism, whereas those with elevated serum TSH butnormal fT4 were regarded as having subclinical hypothyroidism . | Subjects known to have atherosclerotic CVDs (e.g., coronary artery disease, stroke, or peripheral vascular disease), any systemic disease (e.g., DM, chronic renal failure, orchronic liver failure), active or chronic infection,inflammatory diseases, or malignancies were excluded. | NA | 9.6mIU/L |
| Ping Dou | 2016 | SCH: TSH increased and FT4，FT3 was in the normal | Exclusion criteria: (1) patients with hematological diseases, tumors and liver and kidney diseases; (2) Previous or current diseases or thyroid diseases that may lead to hypothyroidism; (3) Recently or taking drugs that affect thyroid function; (4) Pregnant women; (5) Hyperuricemia and hyperlipidemia. | FT3, FT4, TSH: Chemiluminescence method for detection of serum | 4.94mIU/L |
| Qian Jiang | 2020 | SCH: only TSH level increased slightly, while FT4 level was normal, and the patient had no obvious hypothyroidism symptoms. (normal reference value: tsh0. 550-4. 780mlu / ml, FT4: 11. 50-22. 70pmol / L). | Inclusion criteria: (1) in line with the 1999 WHO diabetes diagnostic criteria (2), according to the classification of diabetes mellitus (WHO1999 typing system), the diagnosis was T2DM. (3) The clinical data of the patients were complete. Exclusion criteria: (1) complicated with malignant tumor; (2) Severe organ failure; (3) Pregnant and lactating women; (4) Mental illness; (5) Diseases that significantly affect blood glucose; (6) acute diabetic complications; (7) Previous history of thyroid diseases and recent use of drugs affecting thyroid function; (8) History of hypothalamic or pituitary diseases; (9) The clinical data are incomplete. | NA | NA |
| Qiang Song | 2016 | SCH: TSH increased and FT4，FT3 was in the normal | Selection criteria: all patients met the diagnostic criteria of China guidelines for the diagnosis and treatment of thyroid diseases (revised in 2007); Over 60 years old; Patients with stable condition and clear consciousness who can cooperate with clinical data collection; The patient and his family members signed the informed consent form. Exclusion criteria: Patients with non thyroid diseases causing elevated thyrotropin; Other serious endocrine diseases were treated for the first time; There were hyperlipidemia, hypertension, diabetes, liver disease and nephropathy. Non thyroid diseases causing elevated thyrotropin (TSH); All patients were found without clinical treatment for the first time; Other serious endocrine diseases; Familial hyperlipidemia, hypertension, diabetes, liver disease, and kidney disease have been used for nearly 3 months. They have been used to influence the metabolism of sugar, fat, protein and uric acid (UA). | FT3, FT4 and TSH: measured by microparticle fluorescence enzyme immunoassay.  Uric acid: measured by automatic biochemical analyzer and supporting reagents | 9.7mIU/L |
| Qiannan Du | 2018 | SCH: Serum TSH level is higher than the upper limit of the normal reference value, serum FT3 and FT4 are in the normal range; the reference range of thyroid function is: FT3 2.30-4.20pg/mL, FT40.89-1.76ng/dL, TSH 0.35-5.50 ulU/ ml. | Exclusion criteria：①Type 1 diabetes, special type diabetes, secondary diabetes, gestational diabetes; ②Suffering from various thyroid diseases except SCH, and taking drugs that affect thyroid function, such as iodine, domperidone, lithium, and amiodarone , Phenytoin sodium, metoclopramide and other drugs that affect thyroid function; ③ adrenal insufficiency, pituitary TSH tumors, etc. affect serum TSH levels; ④ lupus nephritis, glomerular disease, tubulointerstitial disease; ⑤ suffer from severe heart, liver, Kidney disease; ⑥ pregnant or lactating women. | Uric acid: using ADVIA2400 automatic biochemical analyzer  FT3, FT4, TSH: Determination by CentaurXP chemiluminescence analyzer | 11.49mIU/L |
| Qing Chen | 2010 | SCHyper: FT3 and FTa are in the normal range, TSH<0.27uU/ml.SCH: FT3 and FT4 are in the normal range, and TSH>4.2uU/ml.Reference range of normal value: FT3: 3.1-6.8pmol/L, FT4: 12-22 pmol/L, TSH: 0.27-4.2uIU/ml | Elimination of liver disease, kidney disease, cancer, diabetes and other secondary dyslipidemia diseases, non smokers or past smoking history has quit smoking for more than 6 months, and in the past 3 months did not take the influence of blood lipids and nail work drugs. | TSH, FT3, FT4: Applied electrochemiluminescence method (Roche, Cobase601, produced by Roche Diagnostics Products Co., Ltd.)  Uric acid: the use of automatic biochemical analyzer and supporting reagents (OlympusAU5400, Olympus Co., Ltd., Japan) enzyme colorimetric method. | 0.17mIU/L（SCHyper）  13.06mIU/L（SCH） |
| Rong Huang | 2013 | Hyperuricemia: male SUA>420umol/L, female SUA>357umol/L | Exclusion criteria: ① patients with a history of thyroid disease; ② Taking drugs affecting thyroid function (contraceptives, glucocorticoids, antiepileptic drugs, etc.) within 3 months before enrollment; ③ Pregnant or within 1 year after delivery; ④ Thyroid antibody positive; ⑤ People with a history of drinking. | Uric acid: uricase-peroxidase coupling method  TSH, FT4, FT3: Chemiluminescence immunoassay | ≤0.69mIU/L（SCHyper）  ≥3.67mIU/L（SCH） |
| Rongrong Zhang | 2013 | SCH: TSH increased and FT4，FT3 was in the normal | Exclusion criteria exclude hematologic diseases, diabetes, malignant tumors, chronic kidney disease, diabetes insipidus and other internal and external diseases that affect serum uric acid levels, as well as the use of diuretics, aspirin and other factors affecting uric acid drugs. | TSH, FT4, FT3: automatic chemiluminescence immunoassay analyzer | NA |
| Ruoxi Tang | 2016 | SCH: TSH 0.27 ~ 10.00 mu / L,TT3,TT4,FT3, FT4 was within the reference range. | Inclusion criteria: (1) age 18 ~ 75 years; (2) thyroid function test shows normal thyroid function or subclinical hypothyroidism, that is, thyroid stimulating hormone (TSH) 0.27 ~ 10.00 mu / L, total triiodothyronine (TT3, reference range 1.2 ~ 3.1 nmol / L), total tetraiodothyronine (TT4, reference range 66 ~ 181 nmol / L), free triiodothyronine (FT3, reference range 3.1 ~ 6.8 pmol / L) and free tetraiodothyronine (FT4, reference range 12 ~ 22 pmol / L) were within the reference range. Exclusion criteria: (1) pregnant women; (2) have received angiography or treatment in recent 3 months; (3) have thyroid hormone resistance syndrome; (4) are taking metoclopramide, sulfonamides and interferon α And other drugs that can lead to abnormal increase of TSH; (5) accompanied by serious liver and kidney diseases; (6) previously clearly diagnosed as thyroid diseases. | Uric acid: detection by chemiluminescence method  TT3, TT4, FT3, FT4, TSH: Chemiluminescence | NA |
| Saini V | 2012 | SCH ： TSH — 6.1 to 9.9 μIU/mL with  normal fT4 and fT3 levels | After informed consent, brief clinical history and examination were done to rule out renal disorders, liver disorder, or any other inflammatory condition which would have influenced theparameters under study. For these analyses, we excluded subjects who were receiving concurrent treatment with drugs that could conribute to hypothyroidism (lithium, amiodarone, or iodine), and those receiving antithyroid drugs (methimazole or propylthiouracil), for  hyperthyroidism. | Uric acid ：Synchron CX4 and CX9 fully automated analyzer (Beckman and Coulter,USA) using diagnostic kits by Randox Laboratories (Crumlin, United  Kingdom).  TSH, fT4 and fT3：fully automated  chemiluminescent immunoassay Analyser Access 2 by Beckman and Coulter (USA).  Reference intervals provided by the manufacturerwere TSH 0.34–5.6 μIU/L, fT3 2.5–3.9 pg/mL, and serum fT4 0.6–1.12 ng/dL. The sensitivities of the TSH, fT3, and fT4 were 0.0025 μIU/L, 1 pg/mL, and 0.4 ng/dL, respectively. | 7.615mIU/L |
| Sayari Saba | 2018 | The diagnosis of subclinical hypothyroidism was performed by the physician by interpreting the results of tests based on increased TSH levels based on the age and normal range of T3 and T4. | Patients who were under treatment with levothyroxine and steroids were excluded from the study. Other exclusion criteria were: having proven hypertension, diabetes mellitus, liver disorders, renal disorders, cardiovascular disorders and patients who had malignancy or were under chemotherapy or radiation therapy. | Uric acid ：biochemical methods with Dirui auto-analyzer  TSH, T3, and T4：enzyme-linked immunosorbent assay reader | 8.94mIU/L |
| Shunyou Deng | 2008 | SCH: TSH > 4.8 mIU / L, 10.3 pmol / L < FT4 < 24.5 pmol / L, and the patient had no obvious hypothyroidism symptoms and signs. | All subjects had no history of familial hyperlipidemia, hypertension, diabetes, liver disease, or kidney disease, and had not taken drugs that affect the metabolism of sugar, lipid, protein, and uric acid in the past 3 months. | Uric acid: measured by automatic biochemical analyzer and supporting reagents  TSH, FT3, FT4: radioimmunoassay. | 9.68mIU/L |
| Tianfang Fu | 2016 | NA | Inclusion criteria: ① the age of pregnancy is not less than 20 years old and not more than 40 years old; ② Pregnancy not less than 24 weeks; ③ Singleton pregnancy; ④ No previous immune system diseases; ⑤ No previous renal disease; There was no diabetes in the past. ⑦ No previous hypertension; ⑧ All subjects had informed consent and participated voluntarily. Exclusion criteria: ① gestational age is less than 20 years old or more than 40 years old; ② Pregnancy less than 24 weeks; ③ Non singleton pregnancy; ④ Artificial insemination; ⑤ Thyroid disease before pregnancy; ⑥ Kidney disease before pregnancy; Diabetes before pregnancy. ⑧ Hypertension before pregnancy; ⑨ Severe mental illness before or during pregnancy. | NA | 6.21mIU/L |
| Torkian P | 2020 | TSH level exceeding 4.0 IU/mL in the presence of a normal range of free prohormone thyroxine concentrations without administering drugs for thyroid disease was labeled as SCH. | During history recording, patients treated with steroids, levothyroxine, and drugs that affect kidney functions were excluded from the study. Having proven hypertension, gout, pregnancy, consumption of protein-rich diet, renal disorders, liver disorders, diabetes mellitus, cardiovascular disorders and patients with malignancy or those undergone chemotherapy or radiotherapy were excluded from the rest of our study. | UA：an auto-analyzer (Hitachi Model 7170 analyzer, Tokyo, Japan)  TSH、T4、T3:ELISA | 5.4mIU/L |
| Wei Liu | 2015 | SCH: TSH increased and FT4，FT3 was in the normal | Exclude hypertension, diabetes, acute cerebrovascular disease, acute and chronic liver and kidney insufficiency, acute infection and rheumatic diseases. | TSH, FT4, FT3: Chemiluminescence immunoassay determination  Uric acid: determination of uricase-peroxidase coupling method | 7.01mIU/L |
| Wei Wei | 2019 | SCH:TSH was higher than the normal reference value (0.35 ~ 5.5 mIU / L), FT3 (3.5 ~ 6.5 pmol / L) and FT4 (11.5 ~ 22.7 pmol / L) were within the normal reference value range. | Inclusion criteria: (1) all patients met the diagnostic criteria of T2DM in who 1999 and received systematic treatment; (2) Meet the diagnostic criteria of climacteric syndrome; (3) The total score of female menopausal self-test scale (Kupperman improved score, Ki score) was ≥ 15; (4) The age ranged from 45 to 55 years. Exclusion criteria: (1) acute diabetic complications and severe chronic complications; (2) Patients with acute infection; (3) Recent surgery and trauma; (4) Bilateral ovariectomy or no function; (5) Serious heart, brain, liver, kidney and hematopoietic system diseases; (6) Severe hypertension; (7) Serious mental illness; (8) Hyperthyroidism, subacute thyroiditis or hypothyroidism are being treated; (9) Those who have received sex hormone drugs in recent 3 months | TSH, FT4, FT3: ADVIA Centaur XP automatic chemiluminescence immunoassay analyzer (Siemens, Japan)  Uric acid: Hitachi 7600-110 automatic biochemical analyzer (Hitachi, Japan) | 6.9mIU/L |
| Weina Xu | 2015 | SCH: TSH increased (TSH≥10 mIU/L), TT4 and FT4 normal range. | All study subjects have excluded clinical hypothyroidism, malignant tumors, liver cirrhosis, chronic pancreatitis, positive viral hepatitis markers, low T3 syndrome recovery period, central hypothyroidism, renal insufficiency, pregnancy. | NA | ≥10mIU/L |
| Wen Cao | 2018 | SCH:FT3,FT4 are in the normal range, and the increase of TSH | Exclusion criteria: all subjects excluded: (1) diseases affecting blood uric acid levels, such as primary gout, diabetes, hypertension, kidney disease, etc. ② Use drugs that increase blood uric acid, such as anti tuberculosis drugs, immunosuppressants, diuretics, etc. ③ After thyroid or pituitary surgery. ④ Liver and kidney dysfunction. ⑤ Acute infection. ⑥ Temporary increase of TSH in the recovery period of clinical serious diseases, recovery period of destructive thyroiditis and injection of TSH. | Uric acid: Swiss Roche C8000 biochemical analyzer, enzymatic method  TSH, FT4, FT3: Swiss Roche E601 automatic electrochemiluminescence analyzer. | 0.153μIU/L(SCHyper)  16μIU/L(SCH) |
| Wenhui He | 2016 | Diagnostic criteria of SCH during pregnancy: serum TSH level is higher than the upper limit of pregnancy specific reference value; The levels of serum FT3 and FT4 were within the range of normal reference values. | All pregnant women are requested to have no history of diabetes and thyroid diseases before pregnancy. | TSH, FT4, FT3: using chemiluminescence method | 6.82mIU/L |
| Wenjuan Jiang | 2016 | (1) TSH in subclinical hyperthyroidism group was lower than 0.350 mu · L-1; (2) TSH in subclinical hypothyroidism group is not less than 5.500 mu · L-1; (3) the patient has no obvious clinical symptoms of hypothyroidism or hyperthyroidism; (4) the levels of serum FT3 and FT4 were normal | Inclusion criteria: (1) TSH of patients in the subclinical hyperthyroidism group is less than 0.350 mU·L -1; (2) TSH of patients in the subclinical hypothyroidism group is not less than 5.500 mU·L － 1; (3) No patients in the subclinical hypothyroidism group Symptoms of clinical hypothyroidism or hyperthyroidism; (4) The patient's serum FT3 and FT4 levels are normal; (5) The patient's biochemical examination results and clinical data are complete; (6) The patient is informed of this study and signed an informed consent.  Exclusion criteria: In this study, if the patient meets any of the following criteria, it will be excluded from this study: (1) The patient took drugs that affect blood sugar or blood lipids within 3 months before the consultation; (2) The patient's serum FT3 and FT4 levels are abnormal (3) Combined with hyperlipidemia, diabetes, liver disease, kidney disease and other diseases; (4) In pregnancy; (5) Actively withdraw from this study. | TSH, FT4, FT3: Hitachi 7600 Biochemical Analyzer | ≤0.35mIU/L(SCHyper)  ≥5.5mIU/L(SCH) |
| Wenping Li | 2020 | Sch: serum TSH > reference is the upper limit (97.5th), and serum FT4 is within the reference value range (2.5th ~ 97.5th). | Inclusion criteria: (1) refer to obstetrics and gynecology (8th Edition) for SPE diagnostic criteria; (2) No chronic diseases in internal surgery; (3) Normal diet and stable signs; (4) Singleton pregnancy; (5) No history of thyroid disease; (6) No disseminated intravascular coagulation; (7) There was no history of drugs affecting thyroid function. Exclusion criteria: (1) thyroid disease, hypertension, diabetes and autoimmune diseases before pregnancy. (2) Heart, liver and kidney dysfunction; (3) Take hypoglycemic drugs, insulin, levothyroxine, antithyroid drugs and other treatment; (4) Combined with respiratory and circulatory diseases; (5) Incomplete medical records. | TSH, FT4, FT3: chemiimmunoluminescence method for detection of serum thyroid stimulating hormone  Uric acid: enzyme colorimetry | 5.79mIU/L |
| Wenzhu Yu | 2015 | NA | All the subjects had no family history of endocrinopathy (diabetes, hypertension, coronary heart disease, kidney disease) and liver disease history. They did not take relevant drugs and lipid metabolism drugs in the past 3 months. | Uric acid: Johnson & Johnson VITROS-5600 automatic biochemical analyzer  TSH, T3, T4: Abbott I2000 | 8.3mIU/L |
| Xiaolei Chen | 2018 | SCH: serum TSH > 4.00mu/l, FT3 and FT4 are within the normal range, and there are no obvious symptoms and signs of hypothyroidism. | NA | NA | ≥4mIU/L |
| Xiaoyan Guo | 2019 | SCH:TSH>4.2mU/L（0.27-4.2mU/L）；FT4 was in normal range（10.3-24.5pmol/L） | Inclusion criteria: aged between 20-75 years; It meets the diagnostic criteria of hypothyroidism. Exclusion criteria: 1. Pregnant and lactating women; 2. Thyroid nodules; 3. Recent history of eating high iodine foods and drugs, regulating thyroid function and lipid metabolism drugs; 4. Those suffering from chronic endocrine diseases, immune diseases, serious cardiopulmonary diseases, tumors, etc. | Thyroid function (using Roche kit): TSH>4.2mU/L (0.27-4.2mU/L); FT4 is in the normal range (10.3-24.5pmol/L).  Uric acid (using Mindray kit): Uric acid is at a normal level (male <420mmol/L, female <358mmol/L). | 5.71mIU/L |
| Xin Wang | 2014 | SCH:FT3,FT4 are in the normal range, and the increase of TSH | Inclusion criteria: Meet the diagnostic criteria of the "Guidelines for the Diagnosis and Treatment of Thyroid Diseases in China" by the Endocrinology Branch of the Chinese Medical Association in 2007; all ages> 60 years; patients are in stable condition, have a clear consciousness, and are able to cooperate in the collection of clinical data; patients and their families have informed consent.Exclusion criteria for non-thyroid diseases that caused elevated TSH; all patients were found for the first time without clinical treatment; other serious endocrine diseases; history of familial hyperlipidemia, hypertension, diabetes, liver disease, and kidney disease, taken in the past 3 months Drugs that affect the metabolism of sugar, lipids, protein, and uric acid (UA). | TSH, FT3, FT4: Using microparticle fluorescence enzyme immunoassay (Apidus, USA) | 9.6mIU/L |
| Xuelian Jiang | 2017 | SCH:4<TSH<10mU/L（0.27-4.2mU/L）；FT4 was in normal range. | Exclusive criteria: all patients were excluded from diabetes with acute complications, malignant tumors, abnormal liver and kidney function, past history of thyroid diseases and those who had thyroid function. | NA | 7.28mIU/L |
| Xueqin Wang | 2019 | SCH:TSH>4.0mIU/L（0.27-4.2mU/L）；FT4,FT3 was in normal range | Inclusion criteria: (1) according to the diagnostic criteria of T2DM, TSH > 4.00mu/l, serum free triiodothyronine (FT3) and serum free thyroxine (FT4) were normal in Sch patients; (2) Relevant inspection and laboratory data in our hospital are complete. Exclusion criteria: (1) patients with severe complications; (2) Patients with thyroiditis, hyperthyroidism, subclinical hyperthyroidism, hypothyroidism and other thyroid diseases; (3) Patients with liver, kidney and pancreas dysfunction or serious diseases; (4) Patients with malignant tumors and immune system diseases; (5) Patients with drugs that affect thyroid function. | Uric acid: Kehua Zhuoyue 360 automatic biochemical analyzer | ≥4mIU/L |
| Yahui Liu | 2019 | SCH:FT3,FT4 are in the normal range, and the increase of TSH | Inclusion criteria: (1) T2DM group with normal thyroid function: 112 patients who met the diagnostic criteria of type 2 diabetes and normal thyroid function. (2) T2DM group combined with SCH: 112 patients who met the diagnostic criteria of type 2 diabetes and met the diagnostic criteria of SCH. (3) Patients who meet the above diagnostic criteria and have complete medical records. (4) Patients who voluntarily assessed their physique and signed informed consent. Exclusion criteria: (1) patients with type 1 diabetes or acute diabetic complications; (2) Patients with severe liver, kidney and heart dysfunction; (3) Patients who have taken drugs (such as amiodarone) that can affect nail work in recent 3 months; (4) Patients with thyroid diseases (such as hypothyroidism, thyroiditis, etc.) that have been clearly diagnosed and / or are being treated; (5) Patients with malignant tumors; (6) Patients with incomplete medical records | Thyroid hormones: chemiluminescence method | 5.66mIU/L |
| Yanbin Zhang | 2017 | SCH: ① serum thyrotropin (TSH) was 97.5th higher than the upper limit of pregnancy specific reference value; ② Serum FT4 2.5 ~ 97.5 th. | Exclusion criteria: ① patients had used drugs affecting thyroid function; ② Patients with other immune system diseases; ③ Renal insufficiency or other renal diseases; ④ Obesity. | TSH, FT4: adopt electrochemiluminescence signal integration method  UA: Colorimetry | 6.78mIU/L |
| Yanhua Xi | 2013 | SCH: serum TSH is elevated, FT4 is normal, and TSH elevations caused by other reasons are excluded. | Inclusion criteria: patients who met the above diagnostic criteria; Age 18-75 years old exclusion criteria: patients who do not meet the inclusion criteria; Acute diabetic complications; Other drugs affecting blood glucose (such as glucocorticoid, phenytoin sodium, etc.) have been used within 2 months; Patients with impaired liver function (AST and ALT were 2.5 times higher than the upper limit of normal); Pregnant and lactating women; Recent pathological conditions that can affect the concentration of inflammatory markers (such as acute or chronic infection, tumor); Complicated with other serious diseases (such as heart failure); Subclinical hypothyroidism caused by various thyroiditis, thyroid nodules, thyroid cancer, thyroid adenoma, pituitary and other malignant tumors, drugs, etc. | Uric acid: enzymatic method  FT3, FT4, TSH: Radioimmunoassay | 7.17mIU/L |
| Yating Zhang | 2012 | SCH: TSH > 4.8 mIU / L, FT3 and FT4 were normal, 10.3 pmol / L < free thyroxine (FT4) < 24.5 pmol / L. | NA | UA: Use Olympus AU2700 biochemical analyzer to detect;  TSH, FT3, FT4: adopt Beckman DXI800 instrument for testing | 7.395mIU/L |
| Yin Li | 2016 | SCH :TSH> 4.5 mIU/L, FT3 and FT4 are normal. | Exclusion criteria:(1) Patients with current or previous thyroid diseases, diseases that may cause abnormal thyroid hormone metabolism and taking drugs that may affect thyroid function in the near future were excluded; (2) Pregnancy status; (3) complicated with serious cardiac and renal insufficiency, tumor and other complications. | Uric acid: automatic biochemical analyzer and original kit | 4.5mIU/L |
| Yingchuan Liu | 2017 | SCH:TSH>4.2 Mu/L，but fT3 and fT4 levels within reference ranges | Exclusion criteria: incomplete data; Clear history of thyroid diseases; Treatment with amiodarone, thyroid hormone and glucocorticoid or received radiocontrast agent two weeks before admission; Other systemic diseases known to seriously affect thyroid function. | Uric acid: HITACHI 7180 automatic biochemical analyzer (Hitachi, Japan)  TSH, FT3 and FT4: Roche Cobas  e601 Automatic Electrochemiluminescence Immunoassay Analyzer (Roche, Switzerland) | 6.95mIU/L |
| Yuan Guo | 2018 | SCH:FT3,FT4 are in the normal range, and the increase of TSH | Inclusion criteria: all cases were treated in the obstetric clinic of the General Hospital of Ningxia Medical University until the end of pregnancy, including pregnant women with abortion, premature delivery, cesarean section and normal delivery; Complete case data; Patients who voluntarily receive thyroid function test; There was no obvious complication of internal and external medicine. Exclusion criteria: those who asked for termination of pregnancy; Twin and multiple pregnancies; Patients with serious internal and external diseases affecting pregnancy outcome | Uric acid: Siemens ADVIA 2400 automatic biochemical analyzer and supporting reagents | NA |
| Yuelei Wu | 2020 | SCH:TSH>4.86 Mu/L，but fT3 and fT4 levels within reference ranges | Inclusion criteria: Patients with essential hypertension who are ≥60 years old, have no gender limitation, and have newly discovered or stopped antihypertensive drugs for more than 1 month.  Exclusion criteria: secondary hypertension, hypertensive emergency, sub-emergency, acute inflammatory reaction within 1 month, history of coronary heart disease, acute coronary syndrome, New York Society of Cardiology (NYHA) heart function classification III to IV, Stroke within half a year, the Cockcroft-Gault formula calculates the endogenous creatinine clearance rate ≤45 ml/min, the serum transaminase ≥ twice the normal reference value, the use of hypoglycemic drugs in the past 1 month or any blood glucose ≥ 13 mmol/ in the past 1 week L, clinical hypothyroidism, hyperthyroidism, use of drugs that affect thyroid function in the past 1 year (iodine-containing drugs, lithium preparations, antithyroid drugs, hypothyroid alternative drugs, etc.), symptom relief, stop anti-inflammatory drugs (non-steroids, Glucocorticoid) Subacute thyroiditis within 1 month, endocrine disease of pituitary hypothalamus and adrenal gland, malignant tumor. | Uric acid: Beckman Coulter AU 5800 automatic biochemical analyzer  TSH, FT3 and FT4: Beckman Coulter DxI 800 automatic immunoassay analyzer using chemiluminescence immunoassay | 4.86mIU/L |
| Yuhong Chen | 2015 | SCH:FT3,FT4 are in the normal range, and the increase of TSH | Exclude hypertension, diabetes, liver and kidney diseases, cancer and other diseases. All the participants had not taken drugs that had an impact on glycolipoprotein uric acid metabolism in recent 3 months. | TSH, FT3 and FT4: determined by chemiluminescence immunoassay | 15.24mIU/L |
| Yuqin Yuan | 2016 | SCH:FT3,FT4 are in the normal range, and the increase of TSH | All subjects had no malignancies, liver and kidney diseases, diabetes, and hereditary hyperlipidemia. No drugs affecting blood glucose, blood lipid and blood uric acid metabolism in recent 3 months. | TSH, FT3 and FT4: use electrochemiluminescence method to detect  Uric acid: automatic biochemical analyzer and supporting reagents | NA |
| Zhang J | 2016 | Hyperuricemia was defined as SUA >420 μmol/L in men and  SUA >360 μmol/L in women [24–27]. Four grades of thyroidfunctional states were determined. Grade I: perfect euthyroidism(TSH from 0.3 to 2.5 μIU/mL, normal FT3 and FT4); grade II:euthyroidism (TSH from 2.5 to 5.0 μIU/mL, normal FT3 andFT4); grade III: mild hypothyroidism (TSH greater than5.0 μIU/mL); and grade IV: mild hyperthyroidism (TSH less than 0.3 μIU/mL). | No participant had known thyroid, renal,hepatic, gastrointestinal, or oncological diseases. | UA： auto-analyzer  (Hitachi Model 7170 analyzer, Hitachi, Tokyo, Japan).  TSH,FT4,FT3： fully automated ADVIA Centaur analyzer  (Siemens Healthcare Diagnostics, New Y ork, USA) by chemiluminescent reaction principle. | ≥5mIU/L |
| Zhangxia Cui | 2016 | SCH: TSH ＞3.0mU/L, FT4 and FT3 levels are normal. | Inclusion criteria: Severe preeclampsia patients who were hospitalized and delivered in our hospital with stable physical signs and no diffuse intravascular coagulation (DIC).  Exclusion criteria: Those with a history of thyroid disease, kidney disease, diabetes, hypertension, etc., and those with severe organ failure. | TSH, FT4, FT3: Adopt Abbott Architect 12 000 kit produced by Abbott, USA  Uric acid: Roche DDP automatic biochemical analyzer, using enzyme colorimetry | 6.4mIU/L |
| Zhiling Hao | 2012 | SCH:FT3,FT4 are in the normal range, and the increase of TSH | All of the candidates met the following criteria: (1) no infectious diseases, familial hyperlipidemia, primary gout, malignant tumors, cardiovascular diseases, cerebrovascular diseases, diabetes, blood diseases, and other autoimmune diseases. ② Not taking vasoactive drugs, thyroid function regulating drugs, lipid regulating drugs, immunomodulatory drugs, anti-inflammatory drugs, antihypertensive drugs, proteinuria lowering drugs and hypoglycemic drugs; ③ There are no bad living habits such as smoking and drinking, and the body mass and blood pressure are within the normal range. | Uric acid: Measured with automatic biochemical analyzer and supporting reagents.  T S H, F T 3, FT4: radioimmunoassay | 7.82mIU/L |
| Mei-hsing | 2004 | Hyperuricemia was defined as uric acid7.9 mg/dL or greater.  Based on plasma TSH levels, subjects were categorized  as hyperthyroid if they had one or more TSH value of  0.1 mIU/L, euthyroid if they had persistent TSH values of  0.1 less than 5 mIU/L, subclinically hypothyroid if they  had one or more TSH value of 5–10 mIU/L, and overtly  hypothyroid if they had one or more TSH value of 10–  99 mIU/L. The euthyroid group was the reference group. | The study cohort included 101,137 elderly adults who had received health examinations between 2005 and 2010. Individuals who had CKD at baseline (n = 26,995),had only one examination from 2005 to 2010(n = 30,053), received medication for thyroid disease(n = 907), had missing serum TSH data (n = 421), were younger than 65 (n = 275) or older than 100 (n = 8), and had abnormal TSH levels that returned to normal(n = 1,024) were excluded (n = 59,683) | NA | 5-10mIU/L |
| Zhengxia Di | 2019 | SCH： ＴＳＨ＞４．７８ｍＩＵ／Ｌ，ＦＴ４, ＦＴ３ were in normal range；SCHyper：ＴＳＨ ＜０．５５  ｍＩＵ／Ｌ，ＦＴ４, ＦＴ３ were in normal range. | All selected persons have no history of taking thyroid drugs | Serum TSH, FT3, and FT4 were determined by direct chemiluminescence method, the instrument was Siemens ADVIA Centaur XP, and the reagents were Siemens original reagents.  UA (Uricase-Peroxidase Enzymatic Method) uses Beckman's original reagent kit. | 0.22mIU/L(SCHyper)  6.42mIU/L(SCH) |
| Song Bo | 2010 | SCH:FT3,FT4 are in the normal range, and the increase of TSH | Except for the increase of TSH caused by other reasons, such as pituitary hypothyroidism, hypothyroidism patients who do not receive drug replacement or supplement in place | NA | 13.56mIU/L |
| Quanyu Li | 2013 | SCH: TSH ＞4.22mU/L, FT4 and FT3 levels are normal. | Exclude the following conditions: acute complications of diabetes, malignancy, abnormal liver and kidney function, past history of thyroid diseases, and the use of drugs that affect thyroid function.  ． | TSH,FT3,FT4: Roche Cobas e601 electrochemiluminescence analyzer was used | NA |
